# Supplementary material for: Programmably tiling rigidified DNA brick on gold nanoparticle as multi-functional shell for cancer-targeted delivery of siRNAs
Source: Nat Commun. 2021 May 18;12:2928. doi: 10.1038/s41467-021-23250-5 (PMC8131747; doi:10.1038/s41467-021-23250-5)
Supplement: Supplementary file 1 — Supplementary Information [file 41467_2021_23250_MOESM1_ESM.pdf]

Supplementary Information for

Programmably tiling rigidified DNA brick on gold nanoparticle as multi-functional shell for cancer-targeted delivery of siRNAs

This file includes:

Supplementary Note

Supplementary Tables 1 and 2

Supplementary Figures 1 to 26

Supplementary References

Supplementary Note

Assembly of sticky-YTDB and its structural advantages. Owing to its mechanical rigidity and structural stability, DNA triangle was often used to produce diverse synthetic DNA nanostructures, including 1D [1, 2], 2D [3-5], and 3D [6, 7] hierarchical architectures, for chemotherapeutic agent delivery [8], gene therapy [9], in vivo imaging [10], and spatially ordered arrays of target species [11]. However, the triangular DNA brick has not been used for construction of core/shell-type nucleic acid nanostructures even if the protection of loaded siRNAs from in vivo degradation is an urgent need for gene therapy because of the lack of a feasible technique to tile DNA bricks on spherical surfaces in a highly organized fashion. In this study, to overcome the technical challenge and to further enhance the nuclease resistance [12], via installing a Y-shaped backbone in the center of common DNA triangle and designing sticky ends at the vertexes, the unique sticky-YTDB with an increased mechanical rigidity was developed and employed for the construction of sandwich-type core/shell nanostructure to create a dense and protective shield suitable for the encapsulated siRNAs.

The structural features of sticky-YTDB, serving as the basic structural unit of horizontally-assembled outer layer, endow the shell with the distinct ability to protect the internal oligonucleotide strands from nuclease degradation. To confirm the structural advantages of sticky-YTDB, the other two YTDB analogs with a similar structure were prepared and their nuclease resistance was comparatively evaluated. As shown in Supplementary Figure 1, the stepwise assembly of the three YTDBs is clearly characterized by native PAGE, where sticky-YTDB is also named YTDB-a. For panel a, the bands with the gradually-retarded electrophoretic mobility are seen, indicating the successive hybridization of Strand 1 with Strands 2 and 3 and the final formation of YTDB-a. Similar trend in the gel electrophoretic mobility shift of DNA hybrids is observed in panel b and panel c, demonstrating the successful assembly of YTDB-b and YTDB-c.

The basic structural units of triangle-contained DNA nanoassemblies are illustrated in Supplementary Figure 2. For YTDB-a, the complementary overhangs (green segment) are designed to install aptamers and the single-stranded (ss) Terminal sucker is used to anchor onto ADC-modified AuNP, while Sticky ends (yellow segment) are palindromic fragments capable of hybridizing with each other between different DNA bricks. The structure of YTDB-b is the same as YTDB-a, but without Sticky ends. Similarly, YTDB-c has no Sticky ends, but contains three Terminal suckers (gray segment) capable of hybridizing simultaneously

with the surface-confined ADCs. Additionally, to explore the roles of Y-shaped backbone, we designed a common triangular DNA brick (CTDB) with sticky ends but without the central Y-shaped backbone and Terminal suckers. Although CTDB is incapable of binding to ADC, it doesn't matter because CTDB only was employed as control for evaluating the stability of YTDB-a against serum degradation but not used for constructing siRNA-encapsulated nanostructures owing to its susceptibility to enzymatic degradation.

Nuclease resistance of YTDB and its assembly on nanoparticle surface. Because the Y-shaped conformation of DNA hybrids exhibits the mechanical rigidity and structural stability [13], Y-shaped backbone installed into the center, combined with sticky-end pairing at the vertex, is expected to endow the DNA triangle with additional nuclease-degradation resistance. The resistance ability of YTDB-a against serum degradation is described in Supplementary Figure 3. Clearly, a large portion of ssDNA and dsDNA are degraded within 1 or 2 h-incubation in 10 % FBS, and CTDB without Y-shaped DNA backbone in its center is substantially digested within 8 h. In sharp contrast, all three YTDBs can exist in FBS for over 24 h even though the amount of residual DNA blocks are different from each other, of which YTDB-a possesses the highest serum stability. Precisely speaking, at the 24 h-incubation in FBS, the residual DNA strands of YTDB-a, YTDB-b, YTDB-c, CTDB, dsDNA and ssDNA are 95%, 60%, 59%, 29%, 0% and 0%, respectively. The measured data demonstrate that the combination of Y-shaped backbone-rigidified geometry with the palindromic fragment-based inter-brick interaction highly efficiently protects DNA components from enzymatic degradation.

Then, the assembly of YTDBs onto ADC-modified AuNP was performed, and their assembly efficiency on CS nanostructure was explored via numbering structural units. As shown in Supplementary Figure 4a, the difference in the fluorescence intensity between siRNA/Ap-CS and siRNA/ADC-AuNP suggests the three YTDBs can separately anchor onto ADC-modified AuNPs as expected albeit to different extents. The number of YTDB-a, YTDB-b and YTDB-c estimated from the corresponding fluorescence peaks are 255, 90 and 577 per AuNP, respectively, which is consistent with the trend in their binding affinity for siRNA/ADC-AuNP: YTDB-c (three Terminal suckers) >YTDB-a (one Terminal sucker+ three Sticky ends) >YTDB-b (only one Terminal suckers). Additionally, as described in Supplementary Table 2, the higher melting temperature of three YTDBs after incubation with ADC-AuNPs also verifies their

successful assembly onto the nanoparticles. Besides, quantification of ADCs covalently attached to AuNP was performed as shown in the section of "**Assembly of siRNA/Ap-CS**" in **Methods**, while siRNAs encapsulated in siRNA/Ap-CS nanoparticle was quantified via fluorescence measurement as described in Step 3 and Step 4 of Fig. 3a. Their numbers are about 720 and 420, respectively. The former is roughly consistent with the previously reported values [14]. Hence, the theoretical encapsulation efficacy of siRNA is 58.3 % (estimated from 420/720×100%). Although the latter number is lower than the former possibly because of the electrostatic repulsion between siRNAs and surface-confined ADCs and steric hindrance, the siRNA-loading content is about 14 times larger than the value incorporated in well-known spherical nucleic acid nanoparticles [15]. Taking into account the molecular structure of the three YTDBs and the correlation between their own number and ADC number on the AuNP surface, YTDB-a units are thought to lie-flat on the siRNA/ADC-AuNP after Terminal sucker/ADC hybridization and cross-link to each other via the Sticky ends, forming pseudohexagonal tiles and more complicated nanostructure. Unlike YTDB-a, YTDB-b lies flat and YTDB-c stands sideways on its one side or one vertex. More information is seen in Supplementary Figure 4b and 4c.

In vivo biodistribution and pharmacokinetics. After confirming the strong enzymatic degradation resistance of core/shell nanostructure required for siRNA delivery, the in vivo biodistribution profile and pharmacokinetic behavior in normal mice were explored by fluorescence-based imaging. Supplementary Figure 8a describes the biodistribution of several siRNA formulations at 1 h following post-injection into normal mice, and each group of mice show the fluorescence signal that varies in the intensity in different regions. For the siRNA/Ap-CS group, the fluorescence signal mainly focuses on the midsection. To offer accurate evaluation, the mice were killed and their organs were harvested for fluorescence imaging. As shown in Supplementary Figure 8b, all the siRNA formulations preferentially distribute in the liver rather than in other organs, but there is a significant difference in the fluorescence intensity from the liver between the three siRNA formulations. Supplementary Figure 8c shows the quantitative measurements of siRNA formulations distributed in the livers, confirming the substantial accumulation of siRNA/Ap-CS in the liver compared with naked siRNA and siRNA/ADC-AuNP. These experimental results are consistent with the previous observations [16], demonstrating that siRNAs loaded in the core/shell nanostructure are

protected from the nuclease degradation in systemic circulation and exhibit the enhanced stability. The in vivo pharmacokinetics of siRNA/Ap-CS was also analyzed. As shown in Supplementary Figure 9, the comparative results demonstrate that siRNA/Ap-CS possesses a long blood circulation time. Specifically, its plasma half-life ($t_{1/2}$) is 3.4 and 9.0 times longer than siRNA/ADC-AuNP and siRNA duplex, respectively. It is worth noting that, the liver toxicity is very low even though the accumulation of siRNA/Ap-CS in liver (Supplementary Figure 10).

Moreover, in vivo pharmacokinetics and biodistribution of siRNA/Ap-CS formulation in tumor-bearing mice were also comparatively explored via time-dependent in vivo fluorescence imaging. As shown in Fig. 4a, the naked siRNA and siRNA/ADC-AuNP cannot be detected in tumor sites throughout the time course. In contrast, the siRNA/Ap-CS nanoparticles unambiguously accumulated in tumor sites (highlighted with dotted cycle) and the fluorescence signal can be detected even at 90 min post-injection, indicating the desirable tumor targeting properties and superior in vivo stability. Fig. 4b shows the quantitative contents of siRNAs within tumor sites by fluorescence measurement, implying that the tumor accumulating efficiency of siRNA/Ap-CS is improved at least by 5.6 times compared with siRNA/ADC-AuNP regardless of incubation time. Fig. 4c demonstrates that siRNA/Ap-CS accumulates in tumor site compared with other organs besides the kidney, and Fig. 4d shows that the content of siRNA/Ap-CS in tumor site is 4.1 times higher than that in liver. Since the kidney servers as an excretory organ through which the nanoparticles are able to be excreted into the urine [17], it is reasonable that a considerable amount of siRNA/Ap-CS is detected in the kidney.

Additional information of cells imaging.

Fig. 5a: releasable siPlk1/Ap-CS, which is the expected and efficient siRNA-loaded targeting formulation.

Fig. 5b: unreleasable siRNA (un-siPlk1)/Ap-CS, which is the same as the formulation in Fig. 5a, but AD strand was used instead of ADC strand and PS-AD strand substituted for PS-ADC strand. Thus, the encapsulated siRNA cannot be released by miRNA from the formulation.

Fig. 5c: releasable siPlk1/CS without aptamer, which is the same as the formulation in Fig. 5a but without aptamer. Thus, the formulation cannot specifically recognize target cells and thus is not internalized even if siRNA is able to be released by hybridization with miRNA.

Fig. 5d: the same as (a) but L02 cells were used instead.

Targeted Delivery, Controlled Release and Gene Silencing Activity. AS1411 can specifically recognize the nucleolin overexpressed in cancer cells such as Hela, MCF-7 and A549 [18, 19]. Moreover, as a well-known "oncomir", the miRNA-21 can serve as intracellular stimulus to trigger the cargo release because it is overexpressed in most cancer cells, including the three cancerous cells [20, 21]. Therefore, Hela, MCF-7 and A549 were employed as the cancerous cell models, while AS1411 was installed onto the outermost protective layer of siRNA-incorporated formulation as targeting ligand model. For the controlled release of siRNA after entering the cells, the sequence of anchoring strand was design to be preferentially complementary to miRNA-21 over siRNA, which is illustrated in the upper panel of Supplementary Figure 11a. The nPAGE analysis verifies that miRNA-21 easily competes for ADCs, and the pre-hybridized siRNAs are completely released, which is evidenced by the reappearance of siRNA band and the complete disappearance of ADC/siRNA band in Lane 3 of the lower panel of Supplementary Figure 11a. The proposed controlled release of siRNAs in response to intracellular stimuli can also occur on AuNP surface (Supplementary Figure 11b). To test the specificity of the response to endogenous stimuli, the fluorescence intensity in the presence of other miRNAs (miRNA-429, miRNA-141 or miRNA-200b) was monitored under identical conditions. As shown in Supplementary Figure 12a, they cannot induce a fluorescence increase except for miRNA-21. Moreover, miRNA-21 can initiate a significant fluorescence change even if incorporating other types of oligonucleotides (DNA and another siRNA) (seen in Supplementary Figure 12b and 12c, respectively) in this nano-formulation. In addition, the stimuli responsive release of siRNAs can occur in FBS solution (Supplementary Figure 12d), indicating the potential application for drug delivery in a complex biological environment. These experimental results demonstrate that the fluorescence signal indeed comes from endogenous miRNA binding rather than the cleavage of fluorophore.

Moreover, as shown in Supplementary Figure 13, the FAM fluorescence signal is easily detected in confocal microscopy images of HeLa cells treated with siRNA/Ap-CS and persists over 10-h period. In

contrast, no obvious signal is achieved with siRNA/ADC-AuNP group from start to finish under identical conditions. This should be because of its insufficient cell permeability since the encapsulated siRNA is able to be displaced by endogenous miRNA-21. The difference in the fluorescence signal demonstrates that the developed siRNA/Ap-CS is capable of performing the targeted delivery of siRNA to cancer cells of interest by actively binding to cell surface receptors for cellular internalization, and the siRNAs can be released by intracellular miRNAs. To further provide convincing evidence, AD and un-siLuc were instead used to prepare unreleasable core/shell nanoparticles as the control of stimulus-responsive release, and the free aptamer-based competitive binding was also conducted. As shown in Supplementary Figure 14a, unlike siLuc/Ap-CS fluorescing efficiently inside cells, un-siLuc/Ap-CS only shows very weak FAM fluorescence, implying that un-siLuc is unable to be released by intracellular miRNA-21 even if un-siLuc/Ap-CS formulations are internalized by target cells. Moreover, as shown in Supplementary Figure 14b, if the cell-surface receptors are pre-blocked with excess free AS1411 aptamers, no obvious fluorescence signal is detected in the confocal image of cells regardless of treating with siLuc/Ap-CS or un-siLuc/Ap-CS, suggesting the two siRNA-encapsulated formulations cannot enter the cells pre-blocked. In addition, the fluorescence cannot be also detected when using the system without aptamer (Supplementary Figure 14c, 14d). This verifies that the cell surface receptor enables the targeted delivery of siRNA-encapsulated core/shell nanoparticle vehicles to specific cancer cells.

More importantly, the high gene silencing activity of siRNA/Ap-CS to suppress luciferase expression was achieved. As shown in Supplementary Figure 15, the siRNA duplex show little effect on the luciferase expression due to its poor stability and inability to enter the cells. The siRNA/ADC-AuNP exhibits weak but detectable gene silencing activity because its cell permeability is poor and the exposed siRNAs transported are only partly protected from endonuclease degradation by AuNP (seen in Supplementary Figure 6). Remarkably, the capacity of siRNA/Ap-CS to suppress the luciferase expression is dramatically higher than siRNA/ADC-AuNP, and the luciferase mRNA was silenced to the level of about 27%, which is comparable with that achieved by Lipo3000, a commercial transfection reagent with a high transfection efficiency that was often employed to carry oligonucleotide strands into diseased cells [22]. The desirable gene silencing activity of siRNA/Ap-CS should be attributed to the combination of its high serum stability (seen in Supplementary Figure 6) and outstanding cell permeability superior to Lipo3000 (seen in Fig. 5e

and 5f, where the mean fluorescence intensity of Cy5-modified siRNAs internalized by the cells was quantified via flow cytometry). To prove the synergistic silencing effects, another siRNA-loaded formulation, siRNA/CS with the protective YTDB coating but without targeting aptamer, was constructed to remain the protection ability against enzymatic degradation but remove the receptor-ligand interaction-enhanced cellular internalization [23]. Its gene silencing efficacy was assessed under identical conditions. As expected, the ability of siRNA/CS to reduce gene expression in HeLa cells is just between siRNA/ADC-AuNP and siRNA/Ap-CS (seen in Supplementary Figure 15), indicating that the absence of aptamer does compromise the gene silencing efficacy.

Supplementary Tables

Supplementary Table 1. The sequences of nucleic acid oligonucleotides designed in this work.[a]

Name		Sequence (5'-3')
**Functionalization of AuNPs**	Anchoring DNA (AD)	SH/TT TT**TAT GTTAA ACCAA GCAAG** TT*TAC TCTCT TAACT CTC*
	Anchoring DNA partly complementary to miRNA (ADC)	SH/TT TTTAT GTTTC AACATC AGTCT GATAA GCTAT T*TACT CT CTTAA CTCTC*
	ADC-BHQ	SH/TT TTTAT GTTTC AACATC AGTCt/ BHQ2/ GATAA GCTAT T*TACT CT CTTAA CTCTC*
**Luciferase siRNA (siLuc)**	LUC-sense partly complementary to AD (LS-AD)	GCUUG AAGUC UUUAA UUAAdT dT**CUUG CUUGG UUUAA CAUA**
	LUC-sense partly complementary to ADC (LS-ADC)	GCUUG AAGUC UUUAA UUAAdT dTGACU GAUGU UGAAA CAUA
	LUC-antisense (LA)	UUAAU UAAAG ACUUC AAGCdG dG
	LA-FAM	UUAAU UAAAG ACUUC AAGCdG dG/FAM
	LA-Cy5	UUAAU UAAAG ACUUC AAGCdG dG/Cy5
***Plk1* siRNA (siPlk1)**	*Plk1* sense partly complementary to AD (PS-AD)	UGAAG AAGAU CACCC UCCUU AdTdT**CUU GCUUG GUUUA ACAUA**
	Plk1 sense partly complementary to ADC (PS-ADC)	UGAAG AAGAU CACCC UCCUU AdTdTGAC UGAUG UUGAA ACAUA
	Plk1 antisense (PA)	UAAGG AGGGU GAUCU UCUUC AdTdT
	PA-FAM	FAM/U AAGGA GGGUG AUCUU CUUCA dTdT
	PA-Cy5	Cy5/U AAGGA GGGUG AUCUU CUUCA dTdT
**Scrambled siRNA**	Scrambled sense partly complementary to ADC (SS-ADC)	UUCUC CGAAC GUGUC ACGUdT dTGACU GAUGU UGAAA CAUA
	Scrambled antisense (SA)	ACGUG ACACG UUCGG AGAAdT dT
	SA-Cy5	Cy5/A CGUGA CACGU UCGGA GAAdT dT
**EcoRI test**	EcoRI-related half sequence partly complementary to ADC (ES-ADC)	AGTGT C*GAAT T*CATA ATGTT GACTG ATGTT GAAAC ATA/FAM
	EcoRI-related another half (EAH)	CATTA T*GAAT TC*GAC ACT
**Test siRNA**	2'OMe modified tested sense partly complementary to ADC (2'-OMe-TS-ADC)	UCAAG GAACU CGAU (CGUAU)$_{2'OMe}$dTdTGAC UGAUG UUGAA ACAUA
	2'-OMe modified tested antisense (2'-OMe-TA)	(AUACG)$_{2'OMe}$AUCGA GUUCC UUGAdT dT/FAM
	Tested sense partly complementary to ADC	UCAAG GAACU CGAUC GUAUdT dTGAC UGAUG UUGAA ACAUA

	(TS-ADC)	
	Tested antisense (TA)	AUACG AUCGA GUUCC UUGAdT dT/FAM
Rat Caspase-3 siRNA	Caspase-3 sense partly complementary to ADC (CS-ADC)	GCAGU UACAA AAUGG AUUAT TGACU GAUGU UGAAA CAUA
	Caspase-3 antisense (CA)	UAAUC CAUUU UGUAA CUGCT G
YTDB-a	Strand 1 (S1)	actac gtcag CCTCG TCCTT CCTGA TCCTT CAAGA TACCA AGAAA CACAA GAAAC AATAC AAGAG TCTAT AGCAT GCGTT AGCAc tgacg tagt*G AGAGT TAAGA GAGTA*
	Strand 1′ (S1′)	AAGAT ACCAA GAAAC ACAAG AAACA ATACA AGA*GA GAGTT AAGAG AGT*
	Strand 2 (S2)	CAAGA AACAA TACAA GAAAG GATCA GGAAG GACGA GGGTG CAAAG TGGAG TAAAG TGCAA GATAC CAAGA AACA
	S2-FAM	CAAGA AACAA TACAA GAAAG GATCA GGAAG GACGA GGGTG CAAAG TGGAG TAAAG TGCAA GATAC CAAGA AACA/FAM
	Strand 3 (S3)	CAAGA AACAA TACAA GACAC TTTAC TCCAC TTTGC ACTGC TAACG CATGC TATAG ACCAA GATAC CAAGA AACA
	Linker strand a (Ls-a)	AAAAT GAGAG AATTG AGAGT TCTTG TATTG TTTCT TGTGT TTCTT GGTAT CTTGa ctgat cagt
	Aptamer (Ap, AS1411)	CTCTC AATTC TCTCA TTTTT TGGTG GTGGT GGTTG TGGTG GTGGT GG
	Ap-Cy3	Cy3/C TCTCAA TTCTC TCATT TTTTG GTGGT GGTGG TTGTG GTG GTGGT GG
	DNA target of miRNA-21 (miRNA-21D)	TAGCT TATCA GACTG ATGTT GA
YTDB-b	Linker strand b (Ls-b)	AAAAT GAGAG AATTG AGAGT TCTTG TATTG TTTCT TGTGT TTCTT GGTA TCTTG
YTDB-c	Strand 4 (S4)	CAAGA AACAA TACAA GAGTC TATAG CATGC GTTAG CACCT CGTCC TTCCT GATCC TTCAA GATAC CAAGA AACA
	Linker strand c (Ls-c)	AAAAT GAGAG AATTG AGAGT TCTTG TATTG TTTCT TGTGT TTCTT GGTAT CTTGT *GAGAG TTAAG AGAGT A*
CYTDB	Linker strand d (Ls-d)	AAAAT GAGAG AATTG AGAGT TCTTG TATTG TTTCT TGTGT TTCTT GGTAT CTTGa ctgat cagt
	Linker strand e (Ls-e)	AAAAT GAGAG AATTG AGAGT TACTG TACTC GTTAA GTCAT TAGTT CGATG TCAAa ctgat cagt
	Linker strand f (Ls-f)	AAAAT GAGAG AATTG AGAGT TCGAG TAGCT GTACC TAGTC ATGTC GTGAT GGCTa ctgat cagt
	Strand 5-FAM (S5)	ACTTA ACGAG TACAG TACAA GATAC CAAGA AACA/FAM
	Strand 6 (S6)	TAGGT ACAGC TACTC GATTG ACATC GAACT AATG
	Strand 7 (S7)	CAAGA AACAA TACAA GAAGC CATCA CGACA TGAC
Primers used for qPCR	*Plk1*-forward	AGCCT GAGGC CCGAT ACTAC CTAC
	Plk1-reverse	ATTAG GAGTC CCACA CAGGG TCTTC
	GAPDH-forward	TTCAC CACCA TGGAG AAGGC
	GAPDH-reverse	GGCAT GGACT GTGGT CATGA
	caspase-3-forward	CATGA CCCGT CCCTT GAA
	caspase-3-reverse	CCGAC TTCCT GTATG CTTAC TCTA

[a]Thiolated AD or ADC is used to modify AuNP, and its italicized fragment close to the 3'-end is the hybridization site for the assembly of YTDB outer layer. The bold fragment in AD is complementary to the bold domain in LS-AD or PS-AD, while the underlined fragment in ADC is able to hybridize with the underlined part in LS-ADC, PS-ADC, ES-ADC, SS-ADC, TS-ADC or CS-ADC. The base region with gray background in ADC represents the sequence complementary to miRNA-21 that can trigger the release of pre-hybridized siRNA. The two italicized fragments in ES-ADC and EAH are capable of hybridizing with each other, forming the cleavage site for EcoRI. The boxed segment in S1, S2, S3 or S4 is designed to hybridize with the boxed domain in the linker strand, including Ls-a, Ls-b and Ls-c. Two fragments in S1 in lowercase are the intramolecular complementary sequences that can form a hairpin-shaped structure via self-hybridization. The double-underlined region in linker strands is design to be capable of binding to the double-underlined region of aptamer, while the region in lowercase letters in Ls-a is a palindromic sequence. The fragments in italics in S1 and Ls-c are used to bind to AD or ADC. In the experiments, ADC/siRNA and AD/un-siRNA are used in pairs during preparing core/shell nanoparticles. For the former, siRNA can be released via strand displacement by intracellular miRNA, but un-siRNA of AD/un-siRNA is unable to be displaced inside target cells. For example, for the siRNA/Ap-CS formulation containing ADC and siPlk1 that is prepared via hybridizing PS-ADC with PA, the siPlk1 can be released via the preferential hybridization of ADC with intracellular miRNA-21. But, when AD and un-siPlk1 composed of PS-AD/PA were instead used for the preparation of core/shell nanoparticles, the encapsulated un-siPlk1 is unable to be released within the cells. The siLuc and un-siLuc were designed according to the strategy similar to siPlk1 and un-siPlk1, respectively.

Supplementary Table 2. Melting temperature of the three different YTDBs before and after positioning onto AuNPs.[a]

Sample	YTDB-a	YTDB-b	YTDB-c
YTDB only	64.3±0.3	62.3±6.8	57.5±2.6
ADC-AuNP+YTDB	75.5±1.0	76.5±0.5	73.0±0.5

[a]The melting temperature was measured by PCR instrument (T100TM Thermal Cycler from BIO RAD).

Supplementary Figures

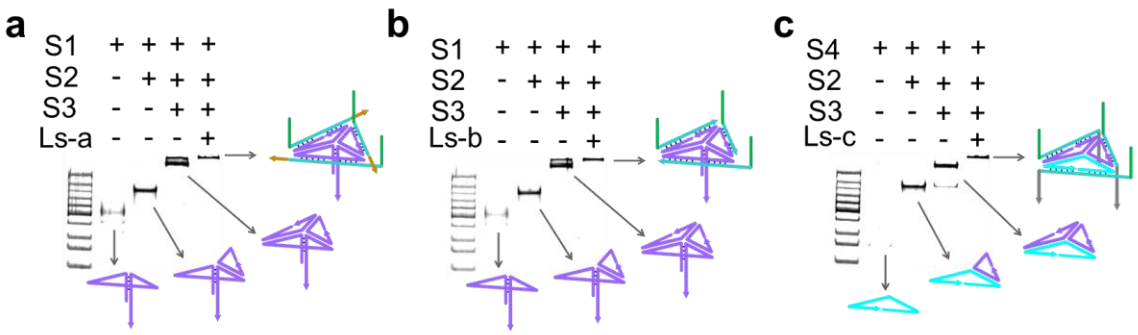

Supplementary Figure 1. Stepwise assembly of three different types of YTDBs. (**a**), (**b**), and (**c**) represent YTDB-a, YTDB-b and YTDB-c, respectively, which are characterized by nPAGE. Strand 1 (S1), Strand 2 (S2), Strand 3 (S3), and Linker strand a (Ls-a) were used for the assembly of YTDB-a. S1, S2, S3, and Linker strand b (Ls-b) were used for the assembly of YTDB-b. S2, S3, Strand 4 (S4), and Linker strand c (Ls-c) were used for the assembly of YTDB-c.

Experimental procedure:

YTDB-a was assembled according to the following procedure: equal amounts (1 µL, 10 µM) of S1, S2 and S3 were added into 19 µL of PBS, followed by heating at 90 °C for 5 min and gradually cooling down to room temperature. Then, 3 µL of linker strand a (10 µM, Ls-a) was injected and allowed to react for 1 h at room temperature. The assembly of YTDB-b or YTDB-c was conducted via employing the same procedure as YTDB-a, but different groups of DNA oligonucleotides were used. Namely, S1, S2, S3, and linker strand b were used for YTDB-b, while S2, S3, S4, and linker strand c were for YTDB-c. To execute the electrophoresis analysis to characterize the as-assembled YTDB, the native polyacrylamide gel electrophoresis (12%, nPAGE) was freshly prepared, and the samples were prepared by mixing 8 µL of YTDB with 2 µL of 6×loading buffer and 2 µL of 10 × Sybr Green I. After loading into the gel wells, nPAGE analysis was conducted at a constant voltage of 80 V in 0.5×TBE (4.5 mM Tris, 4.5 mM boric acid, 0.1 mM EDTA, pH 7.9) on an electrophoresis system (BIO-RAD, USA). The gel images were scanned by ChemiDox XRS Imaging system (Bio-RAD, U.S.A.).

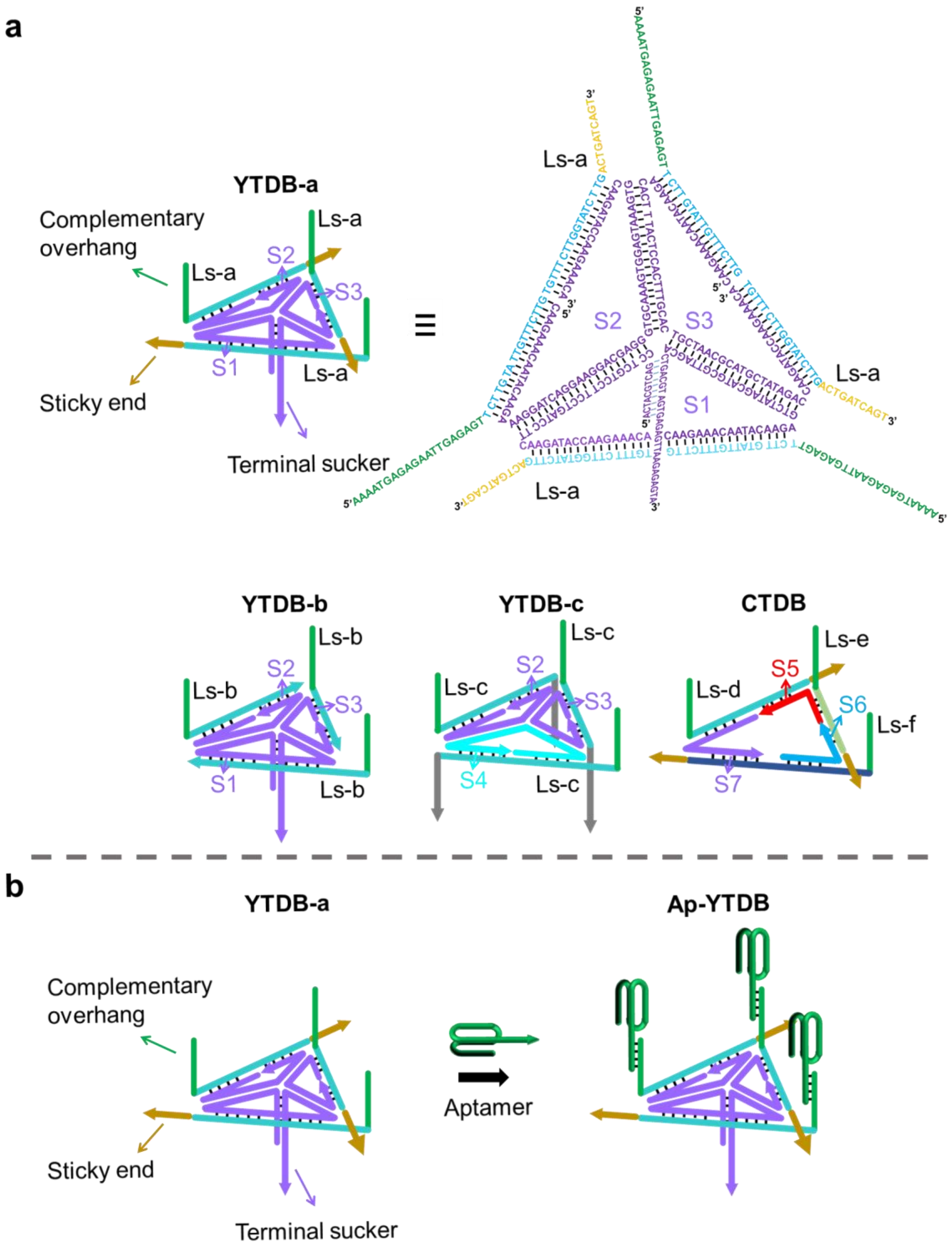

Supplementary Figure 2. (**a**) The schematic illustration of the hybridization among oligonucleotide components for the construction of Y-shaped backbone-rigidified triangular DNA scaffold with palindromic fragment-based sticky ends (YTDB-a) and other control DNA assemblies, including YTDB-b,

YTDB-c and common triangular DNA brick (CTDB). (**b**) The schematic illustration of the binding of AS1411aptamers to YTDB-a.

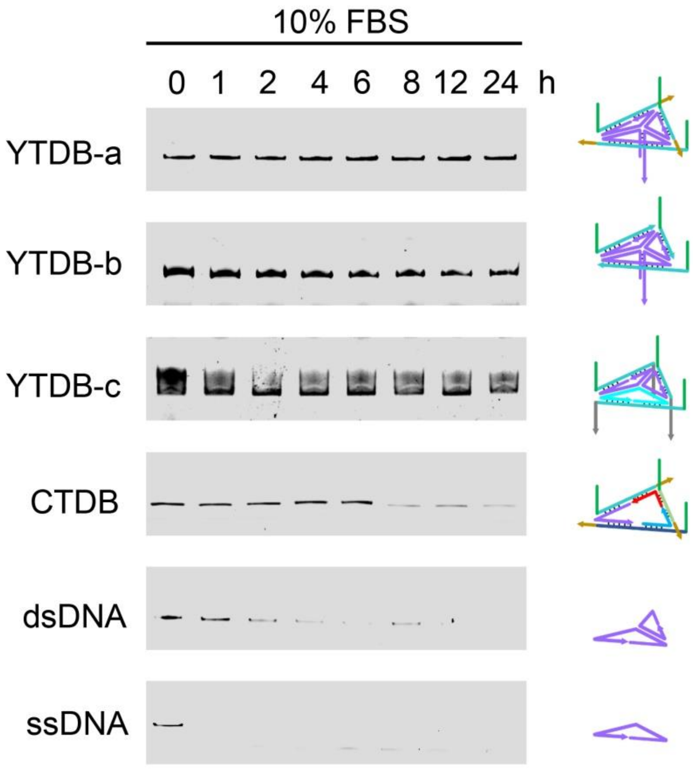

Supplementary Figure 3. Denaturing PAGE (dPAGE) analysis to characterize the resistance ability of YTDB against FBS degradation. S2-FAM, S2 modified with carboxyfluorescein (FAM), was used in this section to fluorescently detect the degradation products. The experiments were conducted three times independently with similar results.

Experimental procedure:

In this section, to conduct the comparative study, six DNA samples were involved: YTDB-a, YTDB-b, YTDB-c, CTDB, dsDNA and ssDNA. A 50-μL aliquot of three different types of YTDBs were prepared according to the method described Supplementary Figure 1. For the assembly of CTDB, a common TDB without Y-shaped DNA backbone, the mixture of equal amounts (2 μL, 10 μM) of Linker strand d (Ls-d), Linker strand e (Ls-e), Linker strand f (Ls-f), Strand 5-FAM, Strand 6 and Strand 7 was added into 38 μL of PBS, followed by heating from 90 °C for 5 min and gradually cooled to room temperature. The double-stranded DNA (dsDNA) sample was prepared via dissolving equal amount (2 μL, 10 μM) of S1 and S2-FAM in 46 μL of PBS, while ssDNA was prepared via adding 2 μL of S2-FAM into 48 μL of PBS. The six

DNA samples were separately mixed with equal amount of d-DMEM (50 μL). All the mixtures were incubated at 37 °C with shaking at 400 rpm, from each of which a 10-μL aliquot was taken out at the time points of 1, 2, 4, 6, 8, 10, 12 and 24 h and stored at -20 °C to terminate the reaction. After mixing with 10 μL of 2×loading buffer, denaturing PAGE (10%, dPAGE) assay was performed in 1×TBE working buffer at a constant current (30 mA) on an electrophoresis system (BIO-RAD, USA). The gel images were scanned by a chemiDox XRS Imaging system (Bio-RAD, U.S.A.).

a

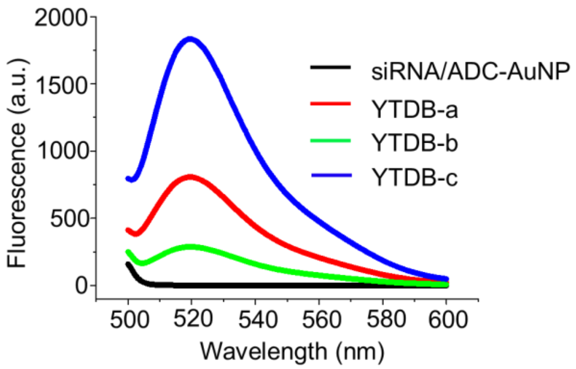

b

**Theoretical value:**	**Measured value:**
Area $_{(YTDB\text{-}a)}$=57 nm^2	$n_{Total\ (YTDB\text{-}a)}$ =254 per AuNP
Area $_{(Hexagon)}$=342 nm^2	$n_{Total\ (YTDB\text{-}b)}$ =90 per AuNP
Surface area $_{(Sphere\text{-}R)}$=16641 nm^2	$n_{Total\ (YTDB\text{-}c)}$ =577 per AuNP
$n_{(YTDB\text{-}a)}$ =83 (monolayer)	
$n_{(YTDB\text{-}b)}$ =287 (monolayer)	N_L=254/83=3 layers

c

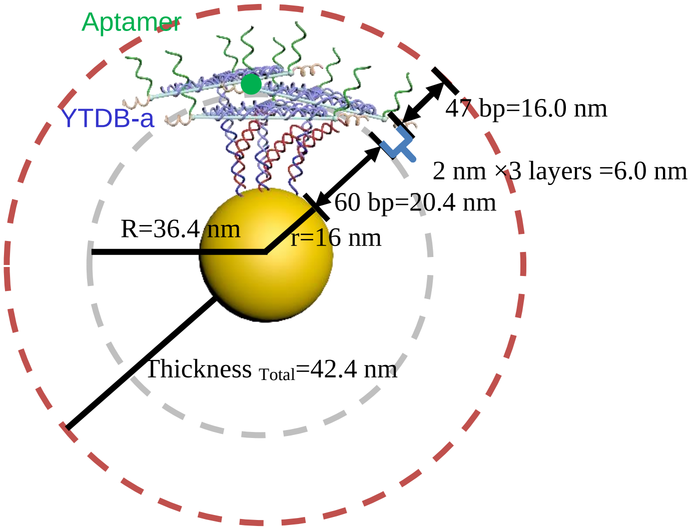

Supplementary Figure 4. Quantitative analysis of YTDBs anchored onto the surface of one AuNP. (**a**) Fluorescence intensity of three YTDBs displaced from AuNP surface. (**b**) The theoretic vales and measured data of the amount of the three YTDBs anchored onto AuNPs. The area of YTDB-a (Area $_{(YTDB\text{-}a)}$), the area of Hexagon (Area $_{(Hexagon)}$), and the surface area of nanosphere (Surface area $_{(sphere\text{-}R)}$) were 57 nm^2, 342 nm^2,

and 16641 nm^2, respectively. The numbers of monolayer YTDB-a ($n_{(YTDB-a)}$) and monolayer YTDB-b ($n_{(YTDB-b)}$) were 83 and 287, respectively. The number of layers (N_L) of YTDB-a on AuNP was calculated by the total number of YTDB-a ($n_{Total\ (YTDB-a)}$) divided by $n_{(YTDB-a)}$. (**c**) The structural analysis of DNA shell, accompanied by the thickness of each layer. The axial distance between adjacent base pairs for the helical pitch and the diameter of double-stranded (ds) DNA are assumed to be 0.34 nm and 2 nm, respectively. The internal layer of 60 pb was obtained from ADC (single-stranded structure with 50 bases) + the 10-bp-contained stem of S1 in YTDB-a (described in Supplementary Figure 2). The length of single-stranded fragments was considered to be similar to the double-stranded structure because the strong electrostatic repulsion on AuNP surface densely coated with oligonucleotides makes them keep in the fully stretched conformation.

Discussion:

Theoretically, the area of one YTDB and total surface area of nanosphere (sphere-R) directly binding to YTDBs are 57 nm^2 and 16641 nm^2 (corresponding to the radius of 36.4 nm), respectively. Along this line, for the YTDB layer on the surface of each AuNP, the number of YTDB-a should be 83 when taking the hexagon void space between the YTDBs into account as illustrated in the middle panel of Fig. 2a, while the YTDB-b number is 287 because there is no the hexagon void space. However, it is not easy to estimate the number of YTDB-c units without palindromic sticky ends because possibly they do not lie flat on the AuNP surface. The actual total amount of the three YTDBs anchored onto AuNPs was estimated by the fluorescence quantitative detection of the supernatant (see details below). The experimentally-measured numbers of YTDB-a, YTDB-b and YTDB-c are 254 (approximately equals to 3×83), 90 (much less than 1×287) and 577, respectively. Taken together, the layer number (NL) of YTDB-a on AuNP is calculated as about 3, while the layer number of YTDB-b is less than 1. Because each YTDB-c has three terminal suckers (each capable of hybridizing with ADC) and the total number of surface-confined ADC is 720 (seen in the section of “**Assembly of siRNA/Ap-CS**” in **Methods**) less than 2×577, the vast majority of YTDB-c bricks should stand sideways onto AuNP surface only via one or two terminal suckers, resulting in the flexible monolayer.

Experimental procedure:

Different AuNP/3D DNA self-assembled layer core/shell nanostructures were prepared as described in the section of "**Assembly of siRNA/Ap-CS**" in **Methods**, whose surfaces were covered with one of three YTDBs (YTDB-a, YTDB-b or YTDB-c). In this section, S2-FAM was used instead of S2 to execute the quantitative analysis by fluorescence measurement. The surface-confined DNA strands were displaced via 24-h incubation with dithiothreitol (DTT) on the basis of a ligand-exchange process. After centrifugation at 12900 g for 25 min, the supernatant containing the released DNA strands was collected, and its volume and fluorescence intensity were immediately measured. Since each YTDB only contains one S2-FAM, the number of YTDB was estimated via quantifying the released S2-FAM. For this propose, the standard curve was pre-established by measuring the fluorescence intensity of free S2-FAM solution at a given concentration ranging from 0, 10, 20, 50, 100 to 200 nM. Taking into account the concentration of AuNP core (0.54 nM) in the core/shell nanostructure solution, the average total number of YTDB-a, YTDB-b and YTDB-c anchored onto the surface of one AuNP is 254, 90, and 577, respectively.

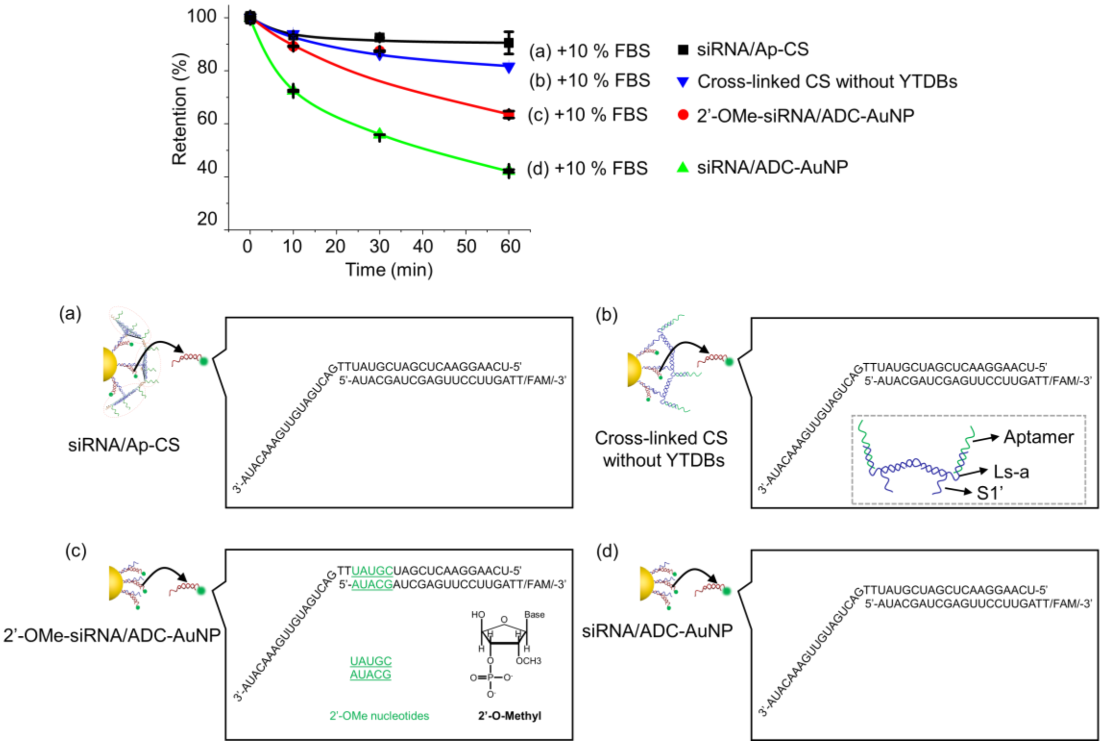

Supplementary Figure 5. Comparative study of the FBS stability of siRNA/Ap-CS with the counterpart nano-formulation under double protection by spherical nucleic acid (SNA) and 2′-O-methyl (2′-OMe) or by cross-linked core/shell nanostructure (cross-linked CS). Quantitative evaluation of the retention (%) of surface-confined siRNAs was performed after exposure of different siRNA-encapsulated formulations (a-d) to FBS, where the retention efficiency of nontreated formulation corresponding to each group is defined as 100%. Nanoparticle-a is the siRNA/Ap-CS with the protective outer coating, Nanoparticle-b is the cross-linked CS, Nanoparticle-c is 2′-OMe-siRNA/ADC-AuNP with the double protective effect of SNA structure and 2′-OMe modification, and Nanoparticle-d is siRNA/ADC-AuNP only with the protective SNA structure. The error bar represents the standard deviation (SD). The measured data are expressed as the means ± SD (n=3).

Discussion:

The residual amount of siRNAs encapsulated in the four formulations decreases in the order of Nanoparticle-a > Nanoparticle-b > Nanoparticle-c > Nanoparticle-d. For example, at the 60-min incubation,

the residual amounts of sRNAs in Nanoparticle-a, Nanoparticle-b, Nanoparticle-c, and Nanoparticle-d decrease to approximately 91%, 82%, 64% and 42%, respectively. These experimental data demonstrate that the FBS stability of siRNA/Ap-CS indeed is much higher than the counterpart formulation, 2′-OMe-siRNA/ADC-AuNP, with SNA/2′ -OMe-based double protection.

Experimental procedure:

The preparation of three siRNA-incorporated formulations (a, c, and d) was performed as described in the section of "**Retention (%) assay of siRNA on AuNPs after treatment with 10% FBS**" in **Methods**. The cross-linked CS was assembled using the same procedure as siRNA/Ap-CS, but S1' was used instead of the hybrid consisting of S1, S2 and S3, where palindromic end-based intermolecular linking effect was preserved but without YTDB. The siRNAs incorporated in Nanoparticle-c were designed by arranging 2′-O-methyl RNA nucleotides at the sites of nuclease hydrolysis according to the literature report [24]. Specifically, the siRNAs in Nanoparticle c were prepared by hybridizing 2′-OMe-TS-ADC to FAM-labelled 2′-OMe-TA), while the siRNAs in Nanoparticle-a (siRNA/Ap-CS), Nanoparticle-b and Nanoparticle-d (siRNA/ADC-AuNP) were 2′-OMe-free siRNAs (TS-ADC and TA). More details on their sequences are shown in Supplementary Table 1.

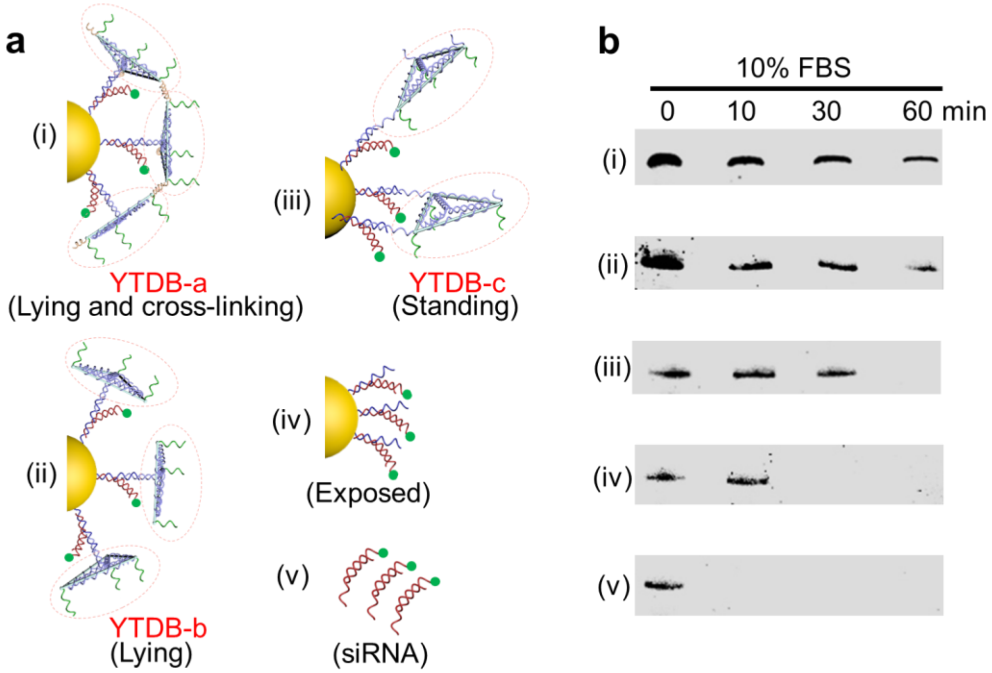

Supplementary Figure 6. The dPAGE analysis to demonstrate the resistance of siRNA/Ap-CS formulation against FBS degradation. (**a**) Schematic illustration of different siRNA-incorporated formulations (Samples i-iv) and siRNA duplex. (**b**) dPAGE images to characterize the residual siRNAs after incubation with 10% FBS. The experiments were conducted three times independently with similar results.

Experimental procedure:

The four siRNA (LS-ADC/LA-FAM duplex where siLuc is modified with FAM)-incorporated formulations used in this section are the same as those mentioned in Fig. 3, and their preparation was described in the section of "**Retention (%) assay of siRNA on AuNPs after treatment with 10% FBS**" in **Methods**. The corresponding siRNA solution and the above siRNA formulations were separately mixed with equal amount of d-DMEM (120 μL) and incubated at 37 °C under stirring at 400 rpm. A 10-μL aliquot was taken out from the resulting solution at the time points of 0, 10, 30, and 60 min, followed by storing at -20 °C to terminate the reaction. Finally, the dPAGE (10%) was conducted to measure the amount of residual siRNAs.

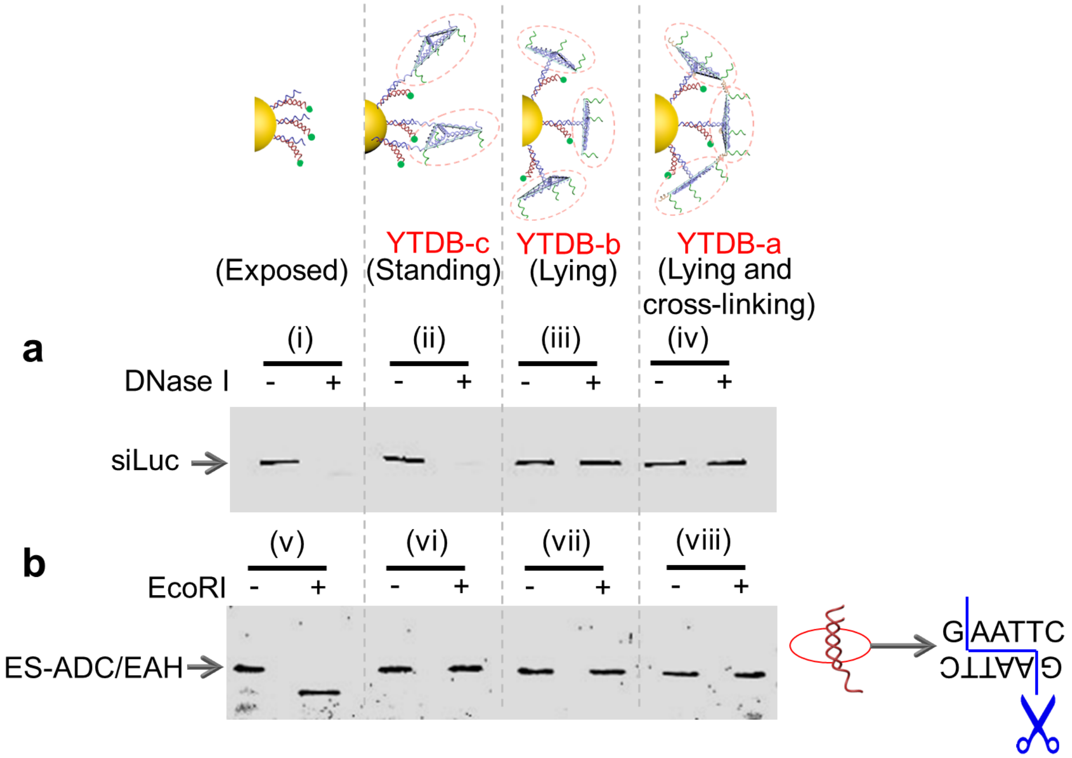

Supplementary Figure 7. Denaturing PAGE analysis to assess the resistance of siRNA/Ap-CS formulation against enzymatic degradation. (**a**) Degradation analysis of siLuc incorporated-formulation after incubating with 100 U/mL DNase I for 30 min. (**b**) The protection of ES-ADC/EAH (EcoRI-related half sequence partly complementary to ADC/ EcoRI-related another half) from restriction enzyme cleavage. The ES-ADC/EAH-incorporated formulation was incubated with EcoRI at the concentration of 150 U/mL for 30 min. The experiments in panels a and b were conducted three times independently with similar results.

Discussions:

The resistance of siRNA-incorporated formulations against an endonuclease that nonspecifically cleaves DNA (DNase I) was first explored. In this section, a double-stranded siLuc consisting of LS-ADC and LA-FAM was loaded during the preparation of core/shell nanoparticle. The measured data demonstrate that DNase I can easily degrade the siRNAs incorporated in ADC-AuNP without the outer layer and core/shell nanoparticles with YTDB-c coating, while no obvious degradation is observed in sample iii with YTDB-b coating and sample iv with YTDB-a coating.

To investigate the resistance of siRNA-incorporated formulations against the cleavage by the restriction endonuclease, EcoRI as a model enzyme was employed and the corresponding restriction site was introduced into a dsDNA prepared via hybridizing EcoRI-related half sequence partly complementary to ADC (ES-ADC) with EcoRI-related another half (EAH). The dsDNA ES-ADC/EAH was loaded into several different formulations. The EcoRI cleavage efficiency is shown in Supplementary Figure 7b. The ES-ADC/EAH in sample v without the protective coating was completely cleaved within 30 min. In contrast, no obvious restriction cleavage occurs in other samples.

Experimental procedure:

For the samples in Supplementary Figure 7a, siLuc consisting of LS-ADC and LA-FAM, which is siRNA silencing luciferase, was used. These core/shell nanostructures can be divided into two groups: one without the protective outermost layer (exposed) and the other with YTDB (YTDB-a, YTDB-b or YTDB-c) outermost layer. The degradation reaction was conducted via incubating the siLuc-incorporated formulation with 100 U/mL DNase I at 37 °C for 30 min. The amount of intact siLuc in the formulation was determined by 10% dPAGE analysis.

For Supplementary Figure 7b, to evaluate the protection of siRNAs from restriction enzyme cleavage, the ES-ADC (EcoRI-sense modified with FAM)/EAH (EcoRI-antisense) duplex (substitute for siRNA)-incorporated formulations were prepared as described in the section of "**Assembly of siRNA/Ap-CS**" in **Methods**. After EcoRI (20000 U/mL) was introduced to reach a final concentration of 150 U/mL, the ES-ADC/EAH-incorporated formulations were allowed to be incubated at 37 °C for 30 min. The resulting solutions were characterized by 10% dPAGE.

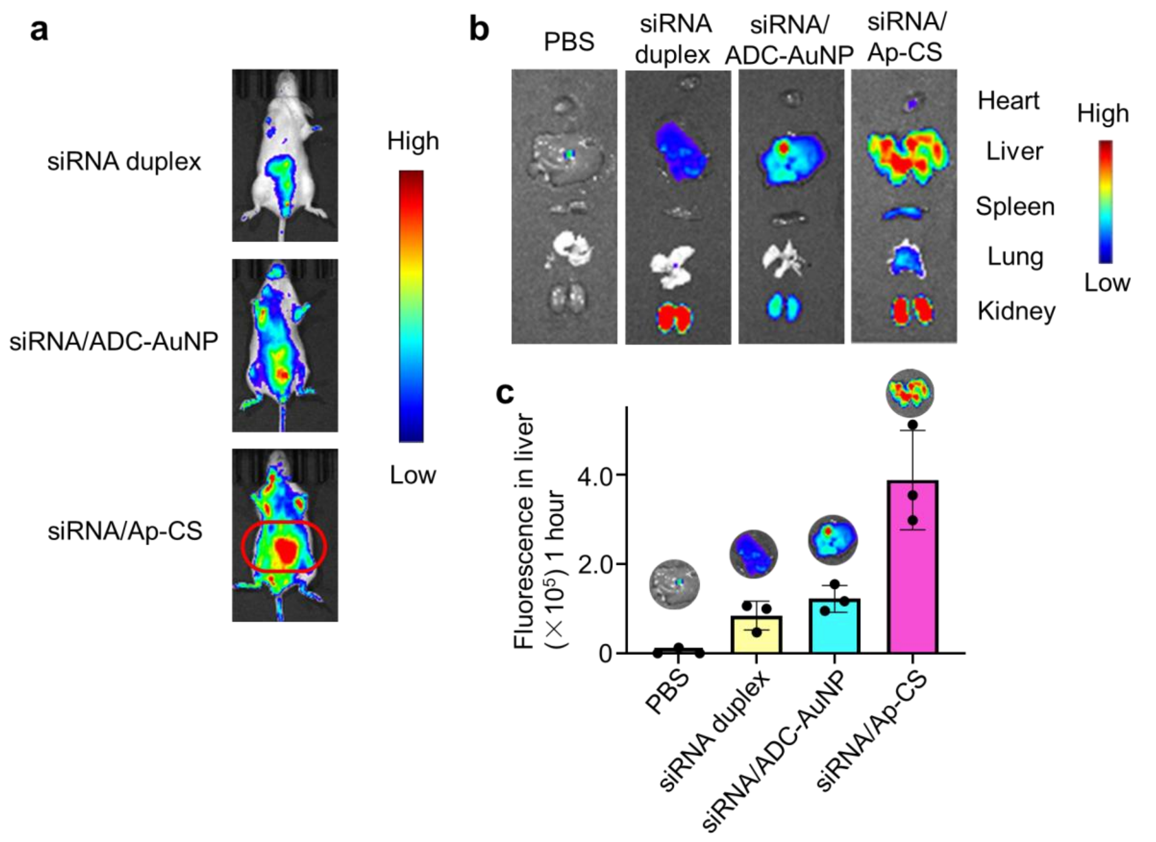

Supplementary Figure 8. Optical imaging to confirm the in vivo biodistribution of siLuc-incorporated formulations. The Cy5-labelled LUC-antisense (LA-Cy5) was used for the preparation of siLuc duplex (LS-ADC/LA-Cy5, LUC-sense partly complementary to ADC/ Cy5-labelled LUC-antisense). (**a**) Fluorescence images of mice after intravenous injection of siRNA duplex, siRNA/ADC-AuNP and siRNA/Ap-CS for 1 h. (**b**) Distribution of siLuc in the organs of mice. (**c**) Fluorescence intensity of siRNA duplex in the livers for different groups of mice recorded under identical conditions, accompanied by the corresponding fields of view. The error bar represents the standard deviation (SD). The measured data are expressed as the means ± SD (n=3).

Experimental procedure:

A 400-µL aliquot of siRNA/Ap-CS solution (LS-ADC/LA-Cy5 hybrid used here) was prepared as described in the section of "**Assembly of siRNA/Ap-CS**" in **Methods**, and the resulting formulation was concentrated to 100 µL in which the final concentration of siRNA is 0.91 µM. Mice (6 weeks old, female) were obtained from Fuzhou Wushi Animal Center (Fuzhou, China). After intravenous injection of

siRNA/Ap-CS (100 μL) for 1 h, the mice were anaesthetized under isofluorane and separately imaged for whole-body fluorescence on Fluorescence Molecular Tomography system (PerkinElmer, U.S.A). Then, the mice were killed and the organs were harvested, followed by the immediate fluorescence imaging. The siRNA duplex and siRNA/ADC-AuNP were used as the controls, and PBS served as blank.

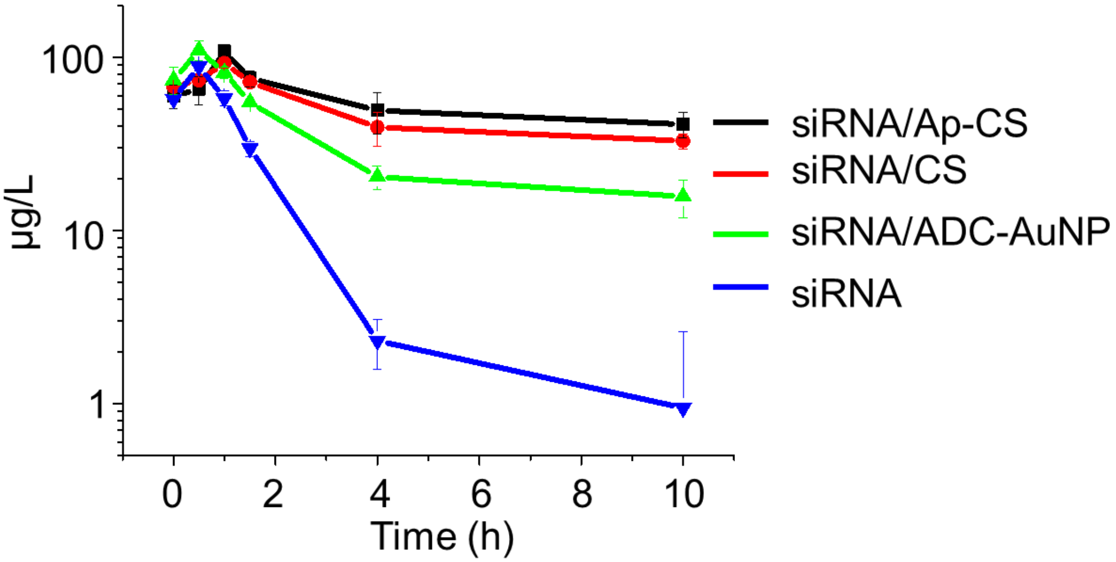

	CL (L/h/kg)	V_{dss} (L/kg)	$t_{1/2}$ (h)
siRNA/Ap-CS	0.13±0.06	0.97±0.28	6.20±3.78
siRNA/CS	0.18±0.06	0.84±0.31	3.81±2.86
siRNA/ADC-AuNP	0.28±0.03	0.65±0.07	1.59±0.22
siRNA	0.59±0.02	0.59±0.03	0.69±0.03

Supplementary Figure 9. The pharmacokinetic profiles of siRNA-incorporated formulations in mice. PA-FAM (FAM-labeled *Plk1* antisense) and PS-ADC (*Plk1* sense partly complementary to ADC) were used for the preparation of siPlk1.The pharmacokinetic parameters in the equations, $t_{1/2}$, CL, and V_{dss}, represent plasma half-life, clearance, and distribution volume at steady state, respectively. The error bar represents the standard deviation (SD). The measured data are expressed as the means ± SD (n=3).

Experimental procedure:

Different siPlk1-incorporated formulations were prepared as described in the section of "**Assembly of siRNA/Ap-CS**" in **Methods.** The siPlk1-incorporated formulation (100 µL, 0.91 µM) was intravenously injected via tail vein. Blood was collected retro-orbitally at the given time point (0, 0.5, 1.0, 1.5, 4 h, 10 h) and centrifuged at 3000 g for 1 min. The plasma (200 µL) was taken from the supernatant and then the concentration of siRNA were measured by fluorescence scanning immediately on a Hitachi F-7000 (Hitachi Ltd., Japan). The standard curve was built to calculate the concentration of siRNA in each blood

sample. The data were analyzed by using the noncompartmental pharmacokinetics data analysis software of DAS version 3.2.2 (Bio Guider Co., Shanghai, China).

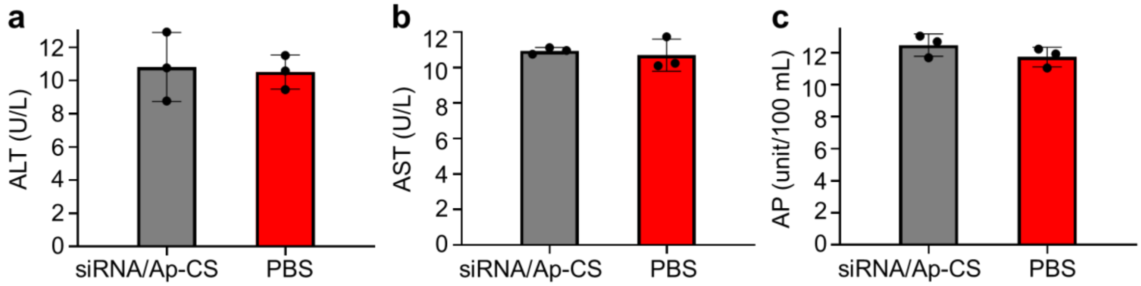

Supplementary Figure 10. The liver toxicity of mice intravenously injected with siRNA/Ap-CS or PBS for 48 h. (a) The alanine aminotransferase (ALT), (b) aspartate aminotransferase (AST) and (c) alkaline phosphatase (AP) were measured by ALT kit, AST kit, and AP kit, respectively. The siRNA/Ap-CS treatment did not cause significant changes in AST, ALT and AP levels, compared with saline controls. The error bar represents in the three panels the standard deviation (SD), and all the measured data are expressed as the means ± SD (n=3).

Experimental procedure:

SiRNA/Ap-CS was prepared as described in the section of "**Assembly of siRNA/Ap-CS**" in **Methods.** The levels of AST, ALT and AP were measured according to the previously-described procedure [25, 26]. Specifically, 100 µL of siRNA/Ap-CS (0.91 µM siRNA) or 100 µL of PBS was intravenously injected via tail vein. After 48 h, the blood was collected retro-orbitally, followed by adding sodium citrate with a mass fraction of 3.2 % (the final volume ratio of 9:1) for clinical chemistry. ALT kit, AST kit, and AP kit were purchased from Nanjing Jiancheng bioengineering institute (Nanjing, China).

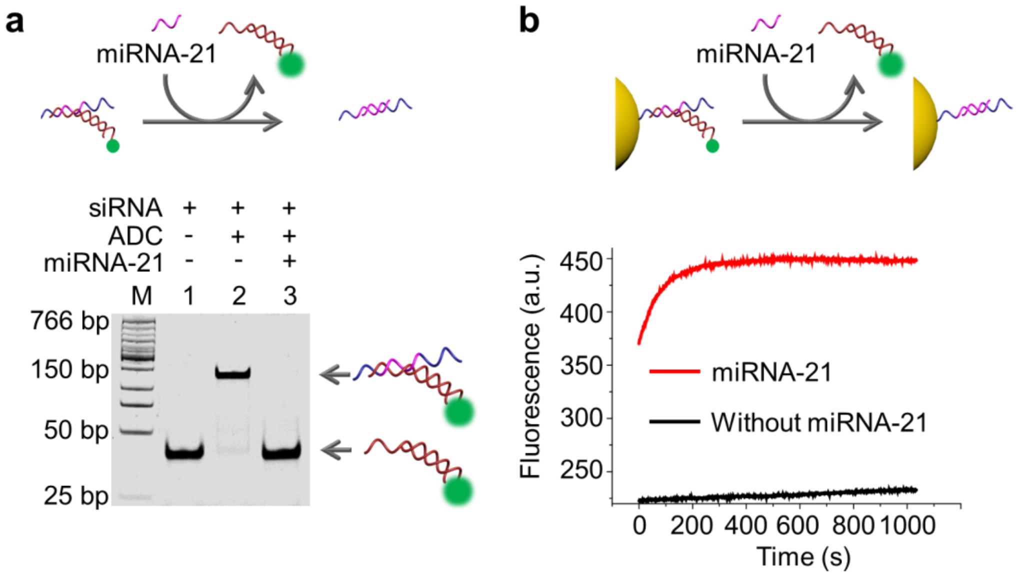

Supplementary Figure 11. Hybridization-based siRNA release from AuNP surface. (**a**) The comparative nPAGE images to demonstrate that miRNA-21D biomarker competes for ADC strand, releasing siRNA duplex (siLuc used here). The experiments were conducted three times independently with similar results. (**b**) The real-time monitoring of fluorescence emission intensity of siRNA/ADC-AuNP in the absence and presence of miRNA-21D. FAM-labeled LUC-antisense (LA-FAM) was used for the preparation of siLuc duplex to monitor fluorescently the hybridization-based strand displacement process.

Experimental procedure:

Panel a: For the sample 3, equal amount of siRNA (LS-ADC/LA-FAM duplex), ADC and miRNA-21D were mixed and incubated at room temperature for 30 min. The DEPC-H_2O was used instead of ADC and/or miRNA-21D for the preparation of sample 1 and sample 2, and the hybridization was allowed to react under identical conditions. The nPAGE was performed at a constant voltage of 80 V in 0.5×TBE on an electrophoresis system (BIO-RAD, USA).

Panel b: The siRNA/ADC-AuNP was prepared as described in the section of “**Assembly of siRNA/Ap-CS**” in **Methods**. After adding miRNA-21D (10 μL, 10 μM) to 200 μL of siRNA/ADC-AuNP, the fluorescence emission intensity of the resulting solution was immediately monitored in real-time. For the control sample, DEPC-H_2O was used instead of miRNA-21D.

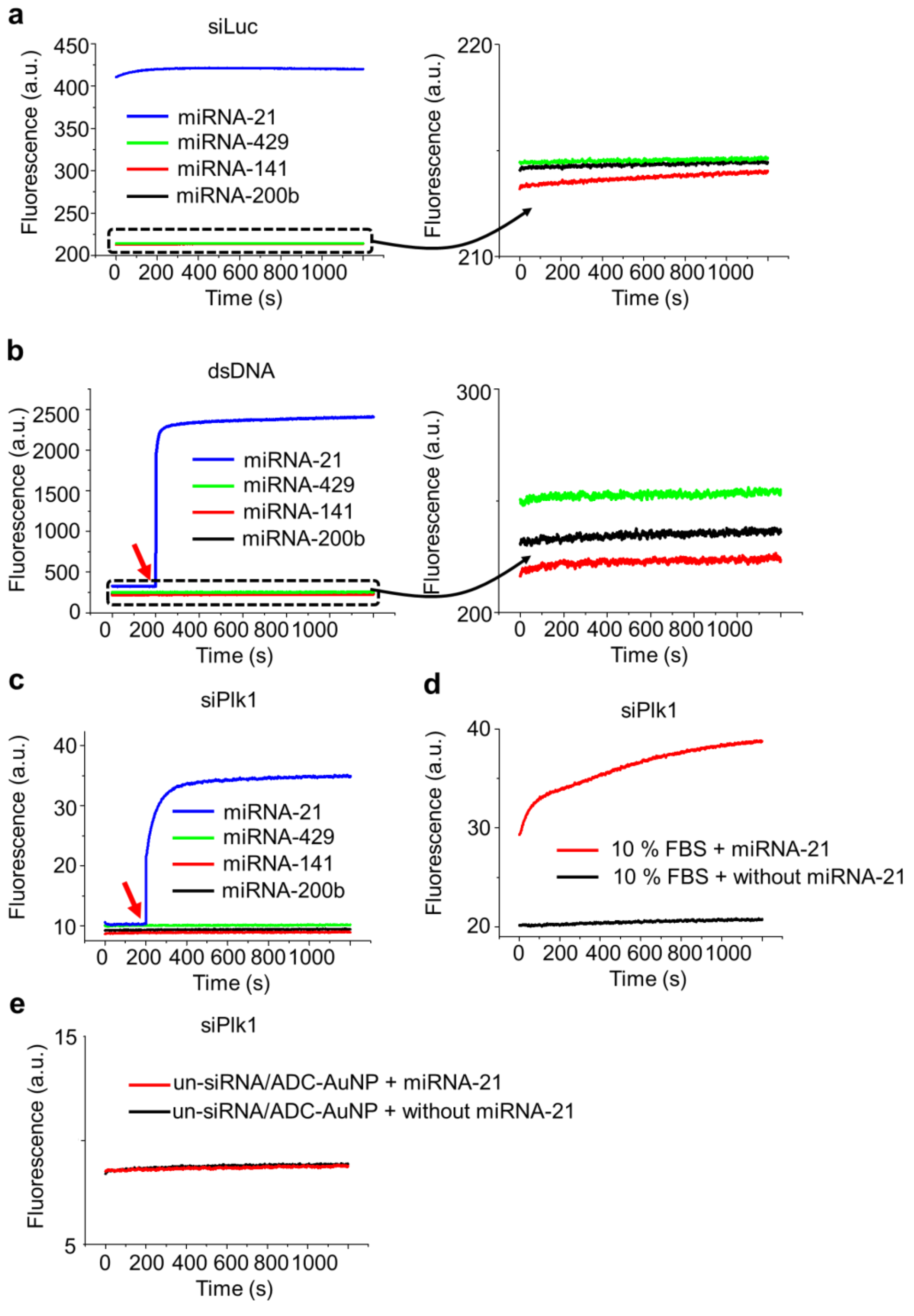

Supplementary Figure 12. (**a**) The real-time monitoring of fluorescence emission intensity of siLuc/ADC-AuNP in the presence of different miRNAs (miRNA-21, miRNA-429, miRNA-141, and miRNA-200b). The siLuc duplex was prepared by hybridizing LS-ADC (LUC-sense partly complementary to ADC) with FAM-labeled LUC-antisense (LA-FAM). (**b**) and (**c**) are the same as (**a**), but siLuc was substituted with

dsDNA for panel b and siPlk1 for panel c. The dsDNA was prepared by hybridizing FAM-labeled ES-ADC to EAH, while siPlk1 was obtained by hybridization of PA-Cy5 (Cy5-labeled Plk1 antisense) with PS-ADC (Plk1 sense partly complementary to ADC). The red arrow at 200 s represents the time point for addition of miRNA stimulus. (**d**) The real-time monitoring of fluorescence emission intensity of siPlk1/ADC-AuNP in the presence and absence of miRNA-21. (**e**) The real-time monitoring of fluorescence emission intensity of un-siPlk1/AD-AuNP in the presence and absence of miRNA-21, where un-siPlk1 was prepared by hybridizing PS-AD with PA-Cy5.

Experimental procedure:

The siRNA/ADC-AuNP (or dsDNA/ADC-AuNP) was prepared as described in the section of "**Assembly of siRNA/Ap-CS**" of **Methods**. While LS-ADC and LA-FAM were used for the preparation of siLuc duplex, FAM-labeled ES-ADC and EAH were used for the preparation of dsDNA, and Cy5-labeled PA and PS-ADC were used for the preparation of siPlk1. After different miRNAs (10 μL, 10 μM) were separately added into 200 μL of siLuc/ADC-AuNP, siPlk1/ADC-AuNP or dsDNA/ADC-AuNP, the fluorescence emission intensity was immediately monitored in real-time.

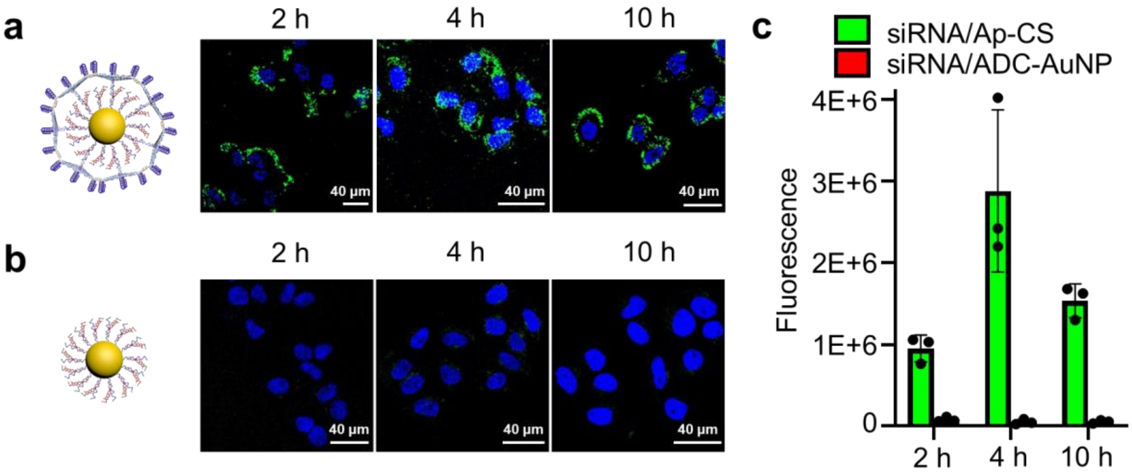

Supplementary Figure 13. Confocal fluorescence imaging of HeLa cells to demonstrate the difference in cellular internalization behavior between siRNA/Ap-CS with AS1411 aptamers (**a**) and siRNA/ADC-AuNP without AS1411 aptamers (**b**). The folded structure in dark blue indicates that aptamer is in its active conformation and thus is prone to interact with the cell receptor. Green fluorescence emission is from FAM covalently attached to siRNA duplex. The fluorescence intensity was measured by Image J software and shown in panel c. The cell imaging in panels a and b was conducted three times independently with similar results, and the measured data are expressed as the means ± SD (n=3). The error bar represents the standard deviation (SD).

Experimental procedure:

HeLa cells were plated on a cover glass (22-mm) in a plastic-bottom plate (12-well) and cultured in DMEM medium containing 10% FBS and 1% penicillin-streptomycin at 37 °C in a humidified atmosphere of 5% CO_2 for 24 h. The siLuc modified with FAM was used here, and the siRNA-incorporated formulation (200 μL) was prepared as described in the section of "**Assembly of siRNA/Ap-CS**" in **Methods**. Before use, siLuc-incorporated formulation was diluted with DMEM to a final volume of 400 μL (the final concentration of siRNA is about 114 nM). After incubating in siLuc-incorporated formulation solution at 37 °C for 4 h, the cells were washed with PBS and then imaged on a Leica SP8 laser scanning confocal microscope (Leica, Germany). The siLuc/ADC-AuNP was used as the control, and the cell imaging was conducted under identical conditions.

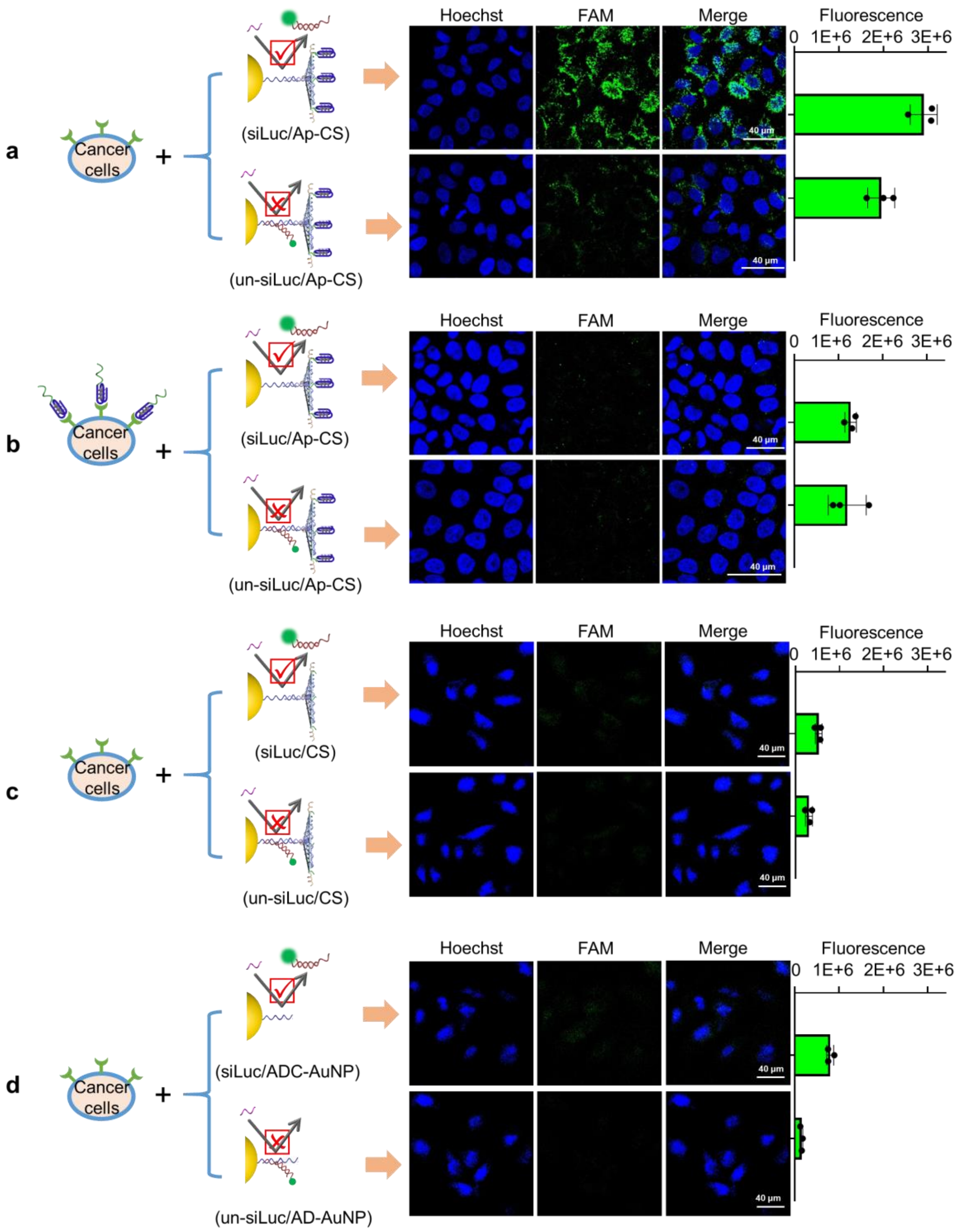

Supplementary Figure 14. Specific membrane receptor-mediated internalization of siRNA/Ap-CS and miRNA-triggered siRNA release, where siLuc is used as the siRNA model. (**a**) Confocal fluorescence imaging of HeLa cells treated with two different siRNA/Ap-CS formulations. *The upper panel:* releasable siLuc/Ap-CS is used, which is the expected and efficient siRNA-loaded formulation. *The lower panel:*

unreleasable un-siLuc/Ap-CS in which LS-AD (LUC-sense partly complementary to AD) strand was used to hybridize with LA-FAM (FAM-labelled LUC-antisense) to prepare fluorescent siLuc, and AD (Anchoring DNA) substituted for ADC strand (Anchoring DNA partly complementary to miRNA). (**b**) The upper and lower panels are the same as the two panels of (**a**) respectively, but HeLa cells were pre-blocked via incubation with excess AS1411. (**c**) The upper and lower panels are the same as the two panels of (**a**) respectively, but siRNA/CS or un-siRNA/CS was instead used. (**d**) The upper and lower panels are the same as the two panels of (**a**) respectively, but siRNA/ADC-AuNP or un-siRNA/AD-AuNP was instead used. The fluorescence intensity was measured by Image J software and shown in the right panel. The error bar represents the standard deviation (SD), and the measured data are expressed as the means ± SD (n=3).

Experimental procedure:

HeLa cells were plated on a cover glass (22-mm) in a plastic-bottom plate (12-well) and cultured for 24 h. The siLuc-incorporated formulations (200 μL) were prepared as described in the section of "**Assembly of siRNA/Ap-CS**" in **Methods** and diluted with DMEM to the final volume of 400 μL (the final concentration of siRNA is about 114 nM). When preparing unreleasable siRNA/Ap-CS, the AD was used instead of ADC, and LS-AD/LA-FAM duplex substituted for LS-ADC/LA-FAM to obtain siLuc. Then, HeLa cells were incubated in the as-prepared siLuc formulation solution at 37 °C for 4 h. After washing with PBS, the cells were imaged on Leica SP8 laser scanning confocal microscope (Leica, Germany). For panel b, HeLa cells were pre-incubated with excess AS1411 to block the nucleolin on the cell membrane.

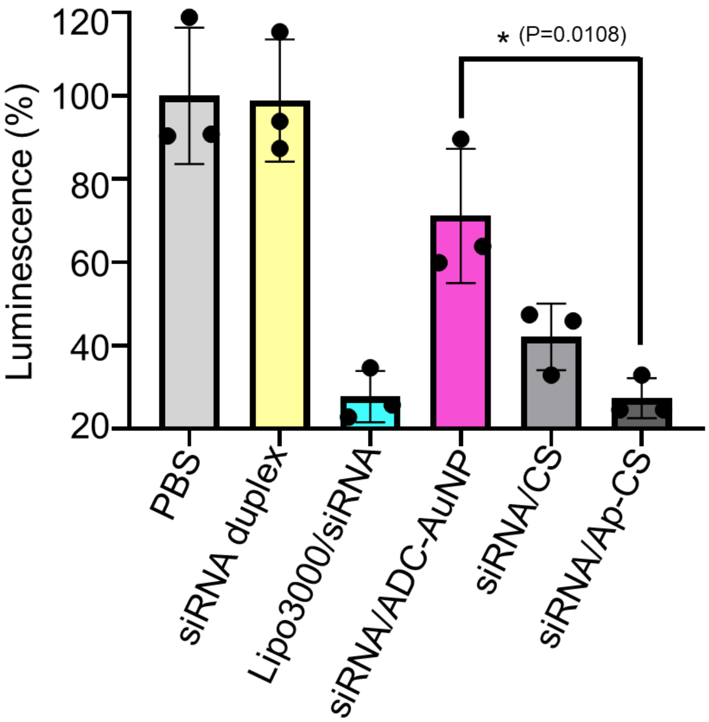

Supplementary Figure 15. Comparative study on the silencing efficacy of luciferase expression in HeLa cells by siRNA/AP-CS formulation. Lipo3000/siRNA indicates that the siLuc is transfected by lipofectamine 3000 transfection reagent. Luciferase activity was normalized to viable cell number treated by PBS under identical conditions. The error bar represents the standard deviation (SD). The measured data are expressed as the means ± SD (n=3). *P<0.05, two-tailed unpaired t test.

Experimental procedure:

HeLa cells were plated on a cover glass (22-mm) in a plastic-bottom plate (12-well) and cultured for 24 h. Various siLuc formulations (200 μL) with releasable siRNA were prepared as the described in the section of "**Assembly of siRNA/Ap-CS**" in **Methods** and then diluted with DMEM to the final volume of 400 μL (the final concentration of siRNA is about 114 nM) followed by incubating with the cells for 48 h. The luciferase expression was measured by Bright-Glo™ Luciferase Assay System (Promega).

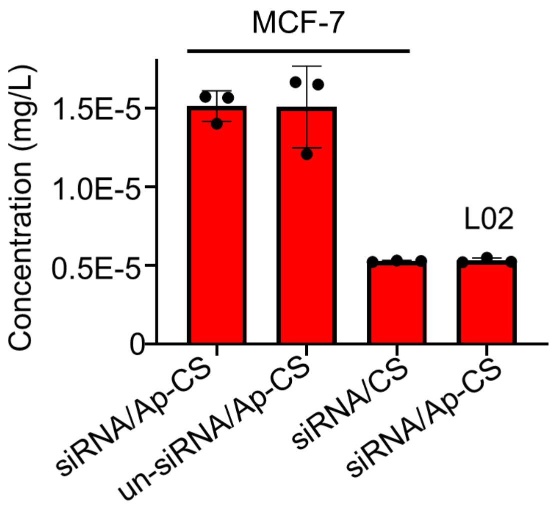

Supplementary Figure 16. The concentration of AuNP per cell in Fig. 5a-d was measured by inductively coupled plasma-mass spectrometry (ICP-MS). The error bar represents the standard deviation (SD). The measured data are expressed as the means ± SD (n=3).

Experimental procedure:

MCF-7 cells were treated with releasable siPlk1/Ap-CS, unreleasable siPlk1 (un-siPlk1)/Ap-CS and releasable siPlk1/CS without aptamer for 4 h. More details of the experiments are offered in the section of "**Cell internalization (Fig. 5)".** The resulting cells were washed with PBS and lysed in acid solution (2% HCl + 2% HNO_3) at 70 °C for overnight. Then, the solution was dilute with ddH_2O to the final volume of 5 mL, and the concentration of gold was measured by inductively coupled plasma-mass spectrometry (ICP-MS, Agilent 7700, America). The concentration of AuNP in L02 treated with siRNA/Ap-CS was detected using the same method.

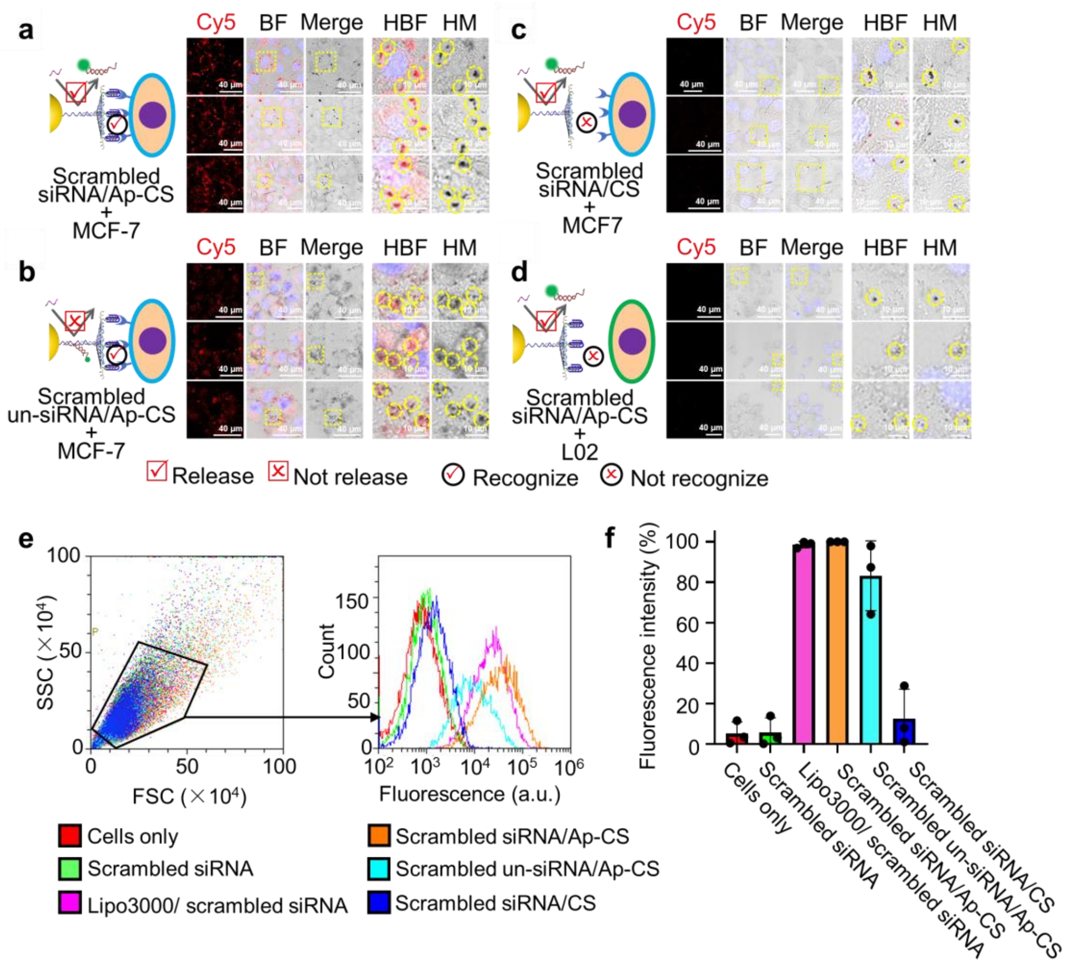

Supplementary Figure 17. Colocalization assay of scrambled siRNA (ssiRNA, red fluorescence) and AuNPs (black dot) within target cancer cells. MCF-7 cells were separately incubated with releasable siRNA/Ap-CS (**a**), unreleasable siPlk1 (un-siRNA)/Ap-CS (**b**) and releasable siRNA/CS without aptamer (**c**) for 4 h. (**d**) is the same as (**a**) but L02 cells were instead used. HM in the right half part is the high-resolution image of the area in yellow dotted box indicated in the section of Merge, while HBF is the high-resolution image boxed in bright field (BF). AuNPs in HM and HBF are highlighted by yellow dotted circles (a-d). These cell imaging experiments were conducted three times independently with similar results. (**e**) Flow cytometry analysis of MCF-7 cells treated with various formulations for 4 h. (**f**) The quantitative fluorescence intensity of each sample in E. The error bar represents the standard deviation (SD). The measured data are expressed as the means ± SD (n=3).

Experimental procedure:

The experiments were the same as Fig. 5, but a scramble siRNA was used instead. The SS-ADC and SA-Cy5 were used for the preparation of siRNA duplex.

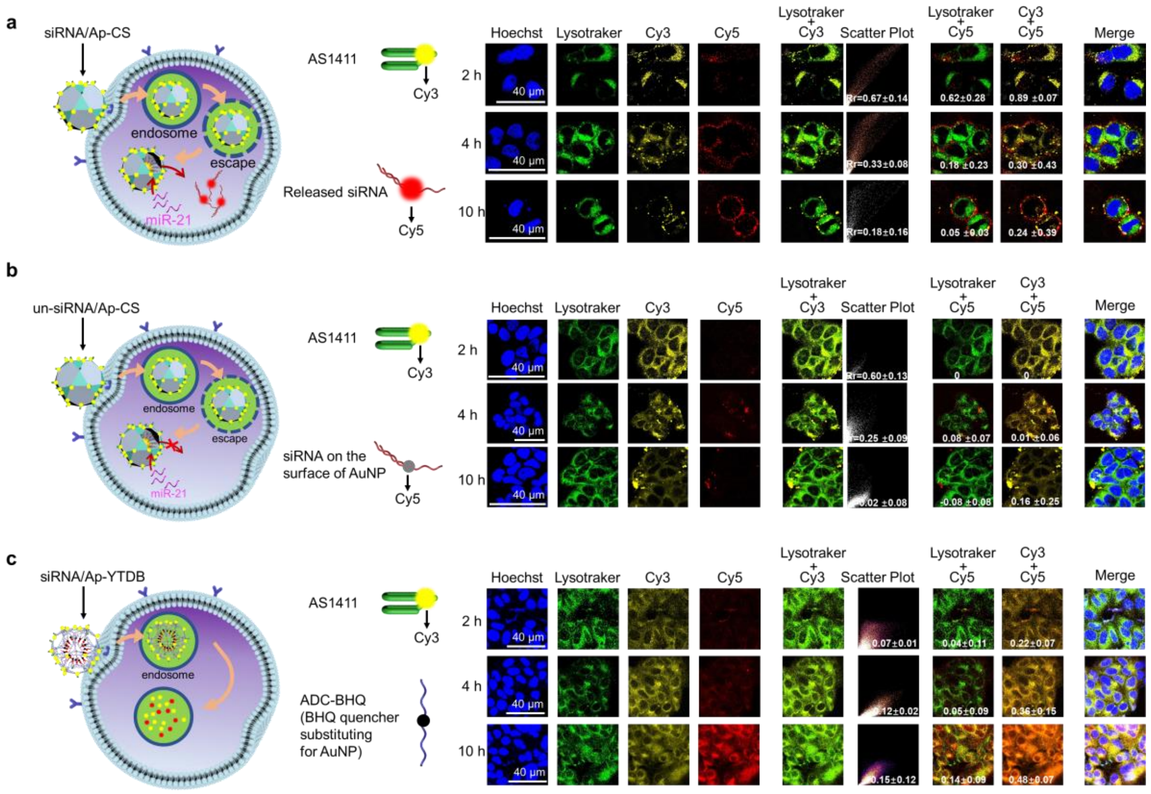

Supplementary Figure 18. The confocal fluorescence images of MCF-7 cells to evaluate the endosome/lysosome escape of siRNA/Ap-CS (**a**), un-siRNA/Ap-CS (**b**) and siRNA/Ap-CS without AuNP (siRNA/Ap-YTDB) (**c**) into the cytosol. The Pearson's coefficient (Rr) was calculated by Image J software. Data were presented as mean ± SD (n = 3).

Discussions

Blue, green, yellow, and red fluorescence represent cell nucleus, lysosome, siRNA/Ap-CS and siPlk1, respectively. The scatter plot of lysotracker fluorescence (a lysosome marker) and Cy3 fluorescence shows a well linear correlation at 2 h, and a high Pearson's coefficient (Rr= 0.67 ± 0.14) is obtained, demonstrating the high spectral overlapping between lysosome and siRNA/Ap-CS fluorescence. However, the scatter plot gradually and significantly shifts after longer-time incubation (e.g., 4 h and 10 h) and the Rr value decreases to 0.33 ± 0.08 and to 0.18 ± 0.16, respectively, indicating the spectral separation of lysotracker and Cy3 [27, 28, 29]. These experimental results demonstrate that siRNA/Ap-CS is firstly delivered into endosomal/lysosomal compartments and then gradually escapes into cytosol where miRNAs exist. The Cy5 fluorescence appears and increases with increasing incubation time and does not overlapped with

lysotracker or Cy3 fluorescence, indicating the release of formulated siPlk1 by endogenous cytoplasmic target miRNAs. For the un-siRNA/Ap-CS used as control, the change of Pearson's coefficient shows a similar trend (Scatter plot) (0.60 ± 0.13 at 2-h incubation, 0.25 ± 0.09 at 4-h incubation and 0.02 ± 0.08 at 10-h incubation), indicating the spectral separation of lysotracker and Cy3. But, no obvious Cy5 fluorescence signal is detected because the siRNA cannot be released by endogenous miRNAs and thus Cy5 fluorescence is always quenched by AuNP. For siRNA/Ap-YTDB used as another control, the change of Pearson's coefficient shows a different trend (Scatter plot) (0.07 ± 0.01 at 2-h incubation, 0.12 ± 0.02 at 4-h incubation and 0.15 ± 0.12 at 10-h incubation), demonstrating the entrapment of siRNA/Ap-YTDB in endosome/lysosome. Moreover, the obvious Cy5 fluorescence signal is detected, indicating the separation of Cy5 from the quencher owing to the degradation of nucleic acids.

Experimental procedure:

Panel a: SiRNA/Ap-CS (siPlk1 used here) was prepared as described in the section of "**Assembly of siRNA/Ap-CS**" in **Methods** and diluted with DMEM to a final volume of 400 μL (the final concentration of siRNA is about 114 nM). To execute the colocalization experiments, PA-Cy5 was used for preparing siPlk1 complex and Cy3-labeled aptamer was arranged onto the outermost layer of DNA shell. MCF-7 cells were plated on a cover glass (22-mm) in a plastic-bottom plate (12-well) and cultured for 24 h. Then, the cells were incubated in siRNA/Ap-CS solution at 37 °C for different time periods. After treating with 100 nM Lysotracker Green for 1 h and washing with PBS, the resulting cells were imaged on a Leica SP8 laser scanning confocal microscope (Leica, Germany).

Panel b: The experiments were performed according to the same procedure as ***Panel a***, but un-siRNA/Ap-CS was used instead of siRNA/Ap-CS.

Panel c: SiRNA duplex (30 µL) was pre-prepared by adding equal amount of (4.5 µL, 10 µM) of PS-ADC and PA-Cy5 into 21 µL of DEPC-treated PBS, followed by fully mixing. YTDB-a (165.5 µL) was assembled according to the following procedure: Equal amounts (4.5 µL, 10 µM) of strand 1 (S1), strand 2 (S2) and strand 3 (S3) were added into 125 µL of DEPC-treated PBS and then heated at 90 °C for 5 min. After gradually cooling to room temperature, 13.5 µL of linker strand a (Ls-a, 10 µM) and 13.5 µL of Ap-Cy3 (10 µM) were added and mixed. The resulting solution was allowed to react for 1 h at room

temperature. Afterwards, siRNA duplex (30 μL), YTDB-a (165.5 μL), and 4.5 μL ADC-BHQ were mixed thoroughly and incubated for 1 h at room temperature. The resulting mixture was diluted with DMEM to a final volume of 400 μL (the final concentration of siRNA is about 114 nM). The colocalization experiments were performed as the description in Panel a.

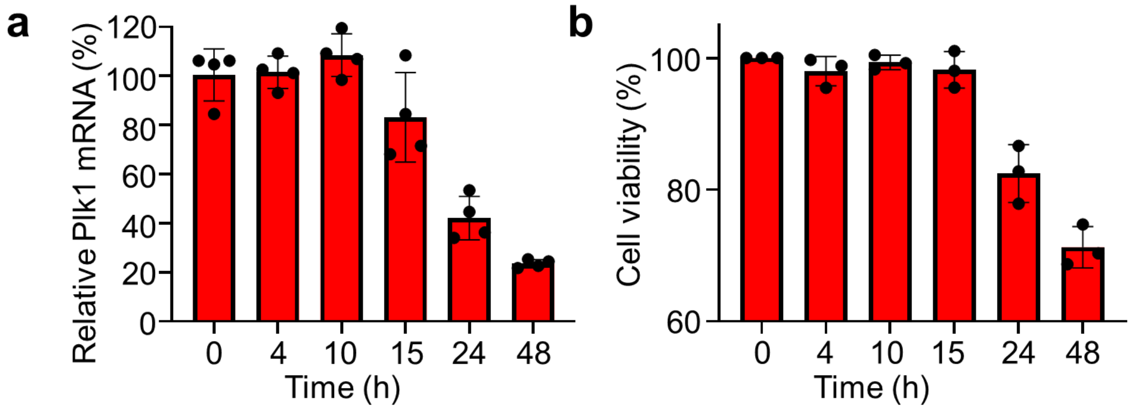

Supplementary Figure 19. The dependence of gene silencing efficiency on the incubating time for treating the cells with siPlk1/Ap-CS. (**a**) *Plk1* mRNA level in MCF-7 cells treated with siPlk1/Ap-CS for different time periods. The measured data are expressed as the means ± SD (n=4). (**b**) The corresponding cell viability. The measured data are expressed as the means ± SD (n=3). The error bar in the two panels represents the standard deviation (SD).

Discussion: Although *Plk1* mRNA level decreases with the incubation time after 10 h, the cell viability almost does decrease until after 48-h incubation. To achieve the high therapeutic efficacy, the 48-h incubation was adopted in the subsequent experiments.

Experimental procedure:

The experiments were performed as described in the section of "**Gene silencing performance of siRNA/Ap-CS formulation in vitro (Fig. 6)**". After treating MCF-7 cells with siRNA/Ap-CS for 0, 4, 10, 15, 24 and 48 h, the *Plk1* mRNA level and cell viability were measured by qPCR and CCK8-kit, respectively.

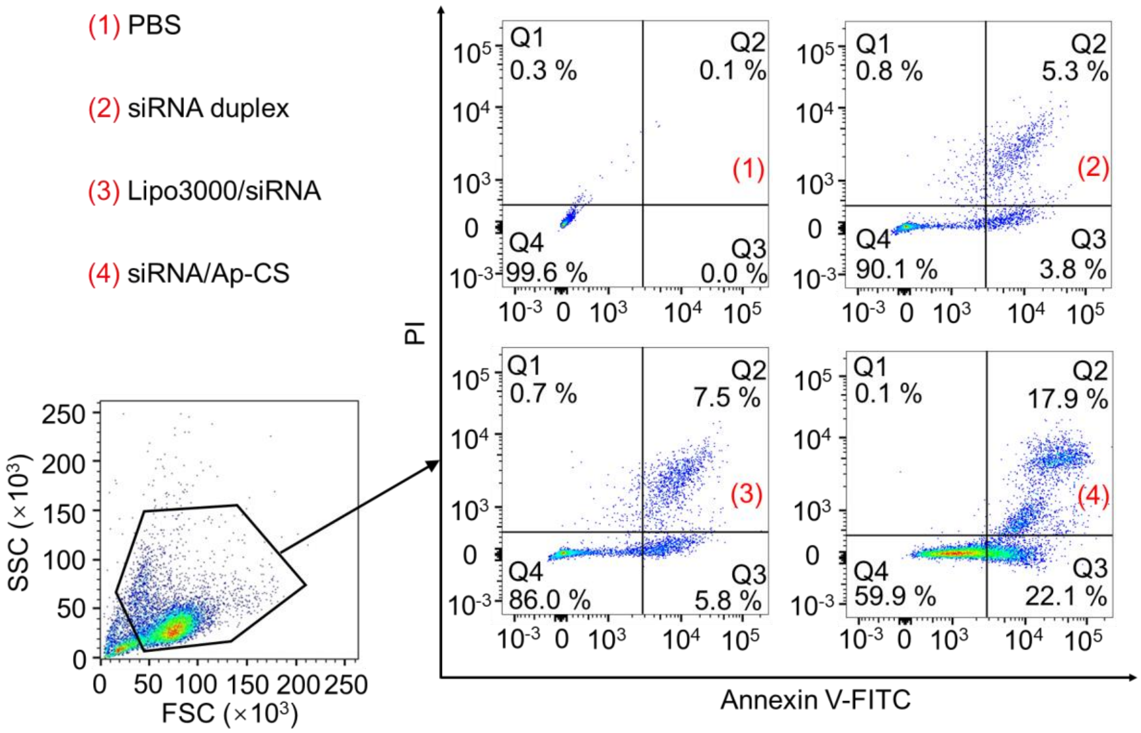

Supplementary Figure 20. Quantitative analysis of MCF-7 cell apoptosis induced by different siPlk1-contained formulations by flow cytometry. Qs 1-4 represent necrotic cells, apoptotic cells in the late phase, apoptotic cells in the early phase and non-apoptotic cells, respectively.

Experimental procedure:

MCF-7 cells were plated on a cover glass (22-mm) in a plastic-bottom plate (12-well) and cultured for 24 h. SiRNA/Ap-CS (200 μL) was prepared as described in the section of "**Assembly of siRNA/Ap-CS**" in **Methods** and then diluted with DMEM to the final volume of 400 μL (the final concentration of siRNA is about 114 nM). Afterwards, the cells were separately incubated with three groups of different siRNA-contained formulations, including siRNA, Lipo3000/siRNA and siRNA/Ap-CS, for 48 h. After executing the Annexin V-FITC/propidium iodide (PI) staining by Annexin V-FITC apoptosis detection kit (Gen-view, scientific Inc., USA), the apoptosis analysis was conducted by flow cytometry. The MCF-7 cells treated with PBS served as the control, and siPlk1 was used as the siRNA model.

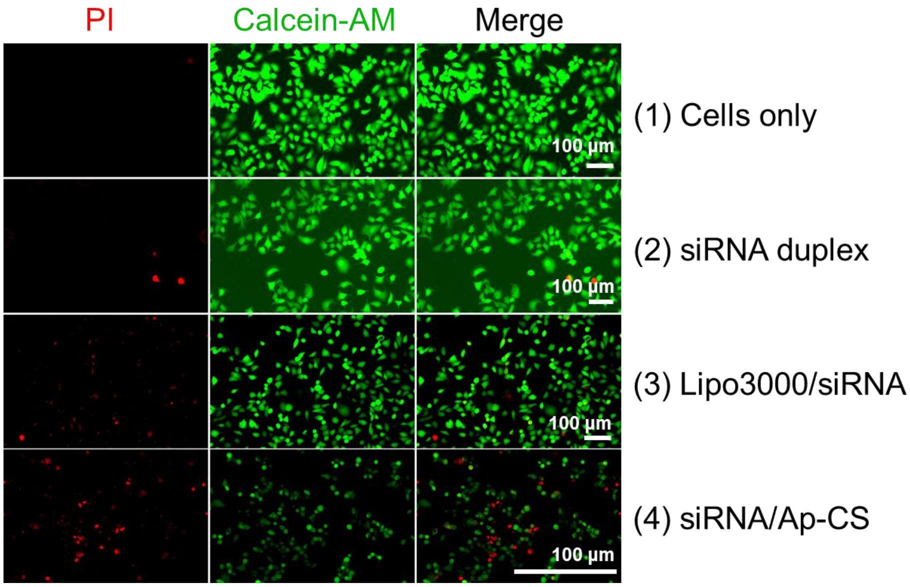

Supplementary Figure 21. Fluorescence microscope images of MCF-7 cells that were stained with calcein-AM and PI after treatment with different siRNA-contained formulations. Green fluorescence and red fluorescence represent living and dead cells, respectively. The cell imaging experiments were conducted three times independently with similar results.

Experimental procedure:

MCF-7 cells were plated on a cover glass (22-mm) in a plastic-bottom plate (12-well) and cultured for 24 h. SiRNA/Ap-CS (200 μL) was prepared as described in the section of "**Assembly of siRNA/Ap-CS**" in **Methods** and then diluted with DMEM to the final volume of 400 μL (the final concentration of siRNA is 114 nM). The siPlk1 was used in this section. The cells were separately incubated with three groups of siRNA-contained solutions for 48 h, and the untreated cells were used as the control. In order to distinguish the dead cells form living cells, calcein-AM/PI staining was carried out according to the instruction of Live/Dead Cell Double Staining Kit. LIVE/DEAD cell assay was executed by fluorescence microscopy.

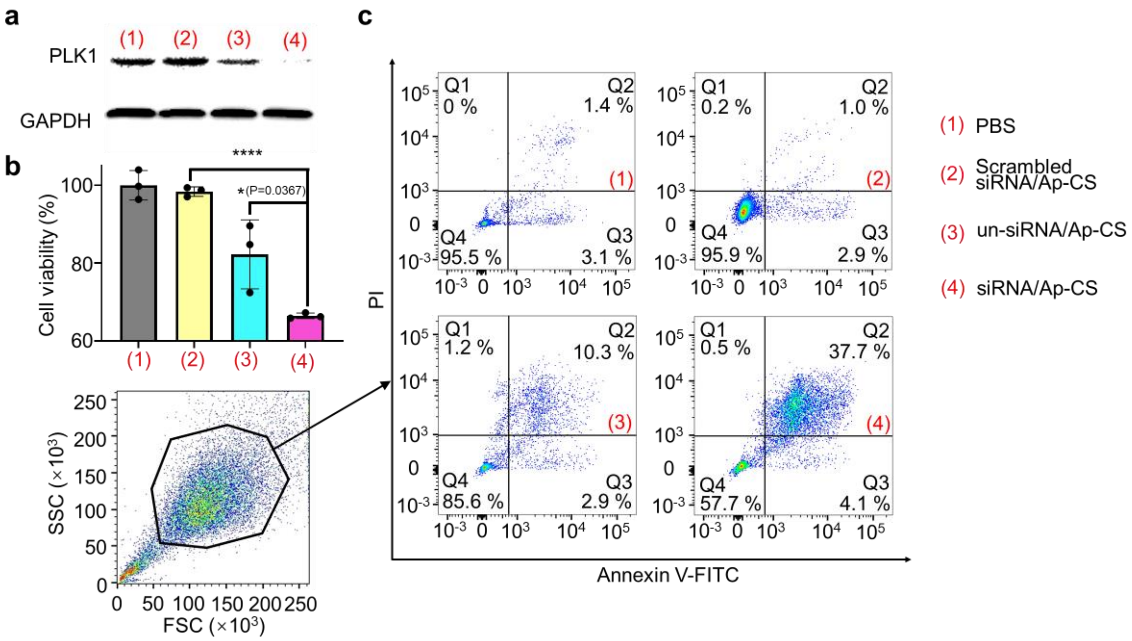

Supplementary Figure 22. The sequence-specific cell apoptosis induced by siRNA/Ap-CS. (**a**) Western blot analysis of PLK1 protein expression in MCF-7 cells treated with siPlk1-incorporated formulation (4, Release) and three controls (1, PBS; 2, Scrambled; 3, Not release). Cell viability (**b**) and apoptosis assay (**c**) of four groups of MCF-7 cells that were the same as (**a**). The error bar in the panel b represent the standard deviation (SD), while the measured data presented are expressed as the means ± SD (n=3). *P<0.05, ****P<0.0001, two-tailed unpaired t test.

Experimental procedure:

The western blot analysis and cell viability assay were conducted according to the procedure adopted in Fig. 6. The apoptosis evaluation was performed by the same procedure as one used in Supplementary Figure 20. The details on the four samples a, b, c and d are as follows:

Sample 1, PBS: PBS was used instead of releasable siPlk1/Ap-CS.

Sample 2, Scrambled siRNA/Ap-CS: the same as Sample d but Scrambled siRNA was used instead of siPlk1 for the preparation of siRNA formulation.

Sample 3, unreleasable siRNA/Ap-CS, which is the same as Sample d but AD strand was used instead of ADC strand and PS-AD strand substituted for PS-ADC strand. Thus, the encapsulated siRNA cannot be released by miRNA from the formulation.

Sample 4, releasable siPlk1/Ap-CS, which is the expected and efficient siPlk1-loaded formulation.

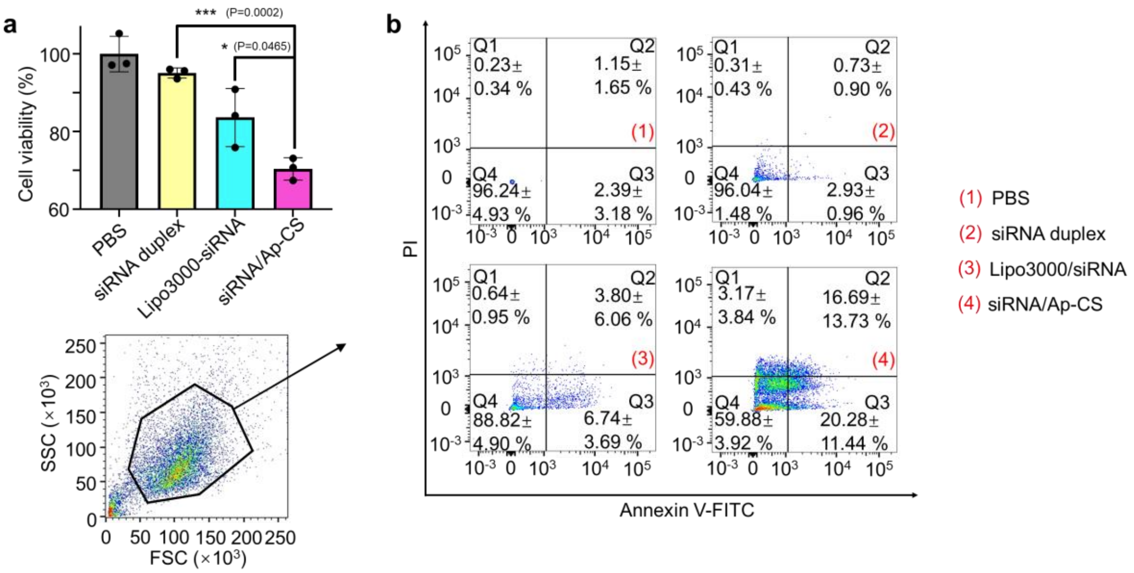

Supplementary Figure 23. Cell viability (**a**) and apoptosis assay (**b**) of A549 cells after treatment with different siRNA-incorporated formulations. Qs 1-4 represent necrotic cells, late apoptotic cells, early apoptotic cells and non-apoptotic cells, respectively. The error bar represents the standard deviation (SD). The measured data are expressed as the means ± SD (n=3 in panel a, n=5 in panel b). *P<0.05, ***P<0.001, two-tailed unpaired t test.

Experimental procedure:

A549 cells were plated on a cover glass (22-mm) in a plastic-bottom plate (12-well) and cultured for 24 h. SiRNA (using siPlk1 as the model)/Ap-CS (200 μL) was prepared as described in the section of "**Assembly of siRNA/Ap-CS**" in **Methods** and then diluted with DMEM to the final volume of 400 μL (the final concentration of siRNA is 114 nM) before sue. The cells were separately incubated with different siRNA-contained formulations for 48 h. The cell viability and apoptosis were analyzed by CCK-8 kit (Sigma-Aldrich, USA) and Annexin V-FITC apoptosis detection kit (Gen-view, scientific Inc., USA), respectively.

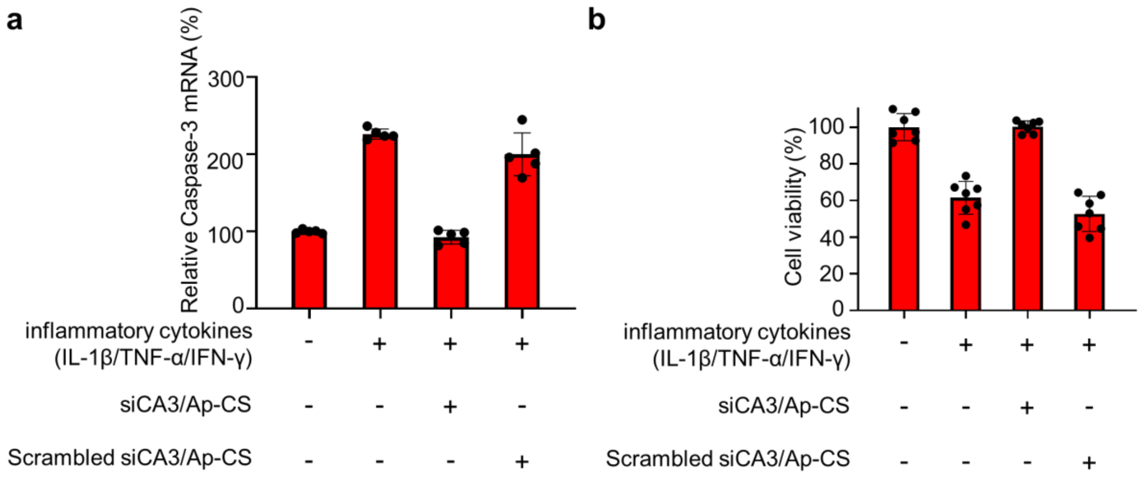

Supplementary Figure 24. The suitability of siRNA/Ap-CS system for silencing caspase-3 protein that causes cell death. After being treated with inflammatory cytokines (25 ng/mL of IL-1β, 5 ng/mL of TNF-α and 25 ng/mL of IFN-γ), the cells were further treated with siRNA/Ap-CS. The relative caspase-3 mRNA levels in rat insulinoma (INS-1E) cells were measured by qPCR (**a**), and the cell viability of INS-1E cells were test by CCK8-kit (**b**). The CS-ADC and CA were used for the preparation of siRNA duplex, and the resulting formulation was called siCA3/Ap-CS. The error bar in the two panels represent the standard deviation (SD), and all the measured data are expressed as the means ± SD (n=5 in panel a, n=7 in panel b).

Experimental procedure:

Up-regulating the expression of caspase-3: The method described in literature [30] was adopted to pre-treat the cells. Specifically, the rat insulinoma (INS-1E) cells, overexpressing miRNA-21 [31], were first plated on a cover glass (22-mm) in a plastic-bottom plate (12-well) and cultured for 24 h. The inflammatory cytokines composed of IL-1β, TNF-α and IFN-γ was diluted with DMEM to the final volume of 400 μL (their final concentrations were 25 ng/mL, 5 ng/mL, and 25ng/mL, respectively). After incubating with the cells for 16 h to increase the caspase-3/7 activity, the inflammatory cytokine solution was removed by pipette and the resulting cells was washed with PBS.

Silencing caspase-3 gene: siCA3/Ap-CS (200 μL) was prepared as the described in the section of "**Assembly of siRNA/Ap-CS**" in **Methods**, but CS-ADC and CA were used for the preparation of siCA3 duplex. Then, siCA3/Ap-CS was diluted with DMEM to the final volume of 400 μL (the final

concentration of siRNA is about 114 nM). After mixing with the above-prepared cells and incubating for 48 h, the caspase-3 mRNA levels were measured by PCR and cell viability of INS-1E was test by CCK-8 kit. Scrambled siCA3/Ap-CS was used as control.

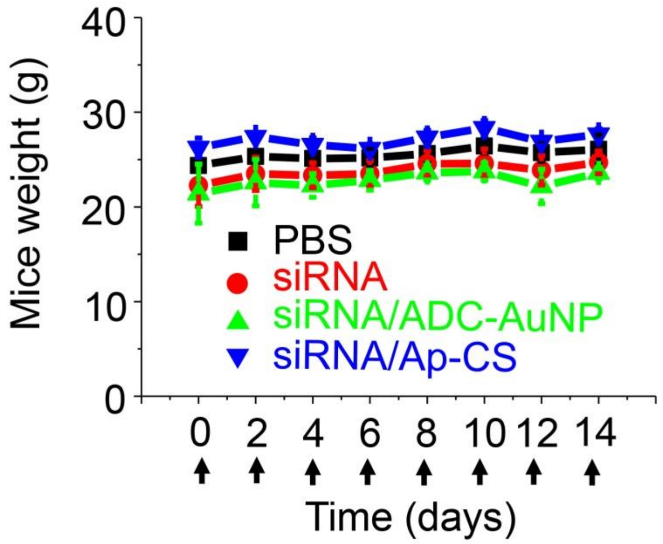

Supplementary Figure 25. Evolution of body weight of each group of tumor-bearing mouse models during the time course of RNAi treatment. Arrows under the number indicate the day of siRNA-loaded formulation injection. The error bar represents the standard deviation (SD), and all the measured data are expressed as the means ± SD (n=3). More details on the experiments are described in Fig. 7.

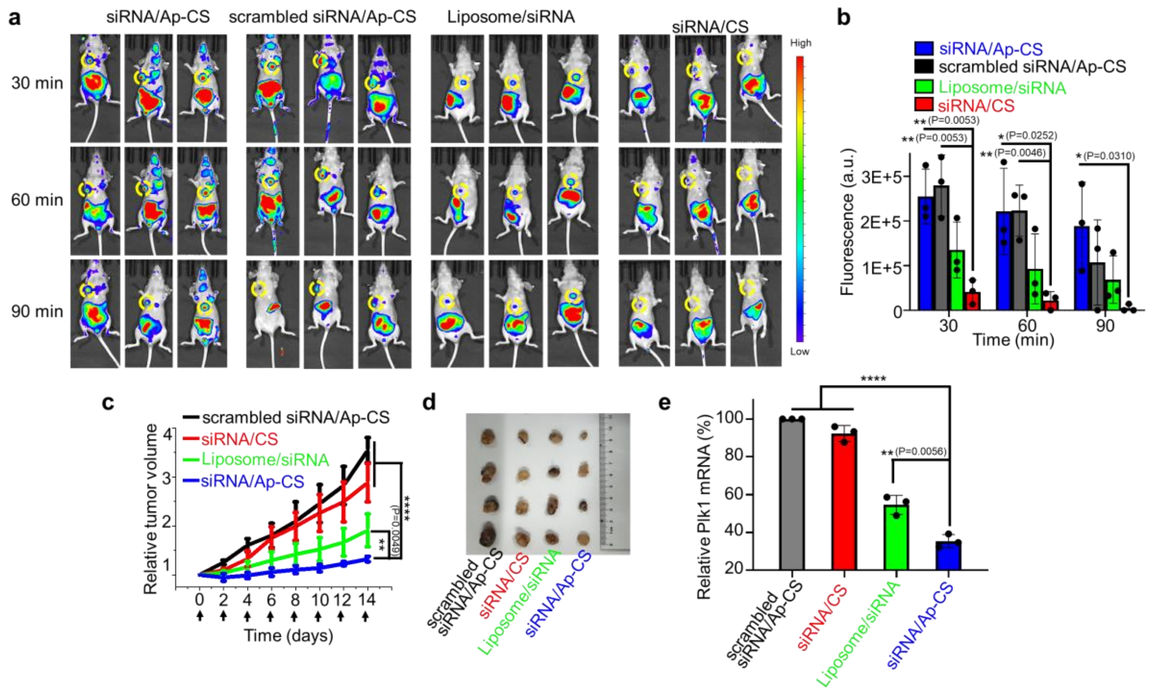

Supplementary Figure 26. In vivo tumor accumulation and therapeutic efficacy of siRNA/Ap-CS against malignant tumor in A549 NSCLC xenograft murine model. (**a**) Time-dependent in vivo fluorescence imaging to explore the whole-body biodistribution kinetics of siRNA/Ap-CS and tumor localization. The samples, including scrambled siRNA/Ap-CS, Liposome/siRNA, siRNA/CS and siRNA/Ap-CS, were administrated to the mice via tail vein injection. Cy5-labeled PA and PS-ADC were used for the preparation of siPlk1. (**b**) The fluorescence intensity of tumors in yellow cycles indicated in (**a**). The error bar represents the standard deviation (SD). The measured data are expressed as the means ± SD (n=3). (**c**) Tumor growth curves during treating tumor with siPlk1 delivered by different formulations. The arrows represent the intravenous injections via tail vein. The error bar represents the standard deviation (SD). The data are presented as the mean ± SD of four independent experiments. (**d**) Images of harvested tumors 14 days after treatment with different siPlk1-encapsulated formulations. (**e**) *Plk1* mRNA levels in harvested tumors were measured by qPCR. The error bar represents the standard deviation (SD). The measured data are expressed as the means ± SD (n=3). *P<0.05, **P<0.01, ****P<0.0001, two-tailed unpaired t test.

Experimental procedure:

Different siPlk1-incorporated formulations were prepared as described in the section of "**Assembly of siRNA/Ap-CS**" in **Methods**, and the A549 tumor-bearing mouse models were established as described in the section of "**Gene silencing performance of siRNA/Ap-CS formulation in vivo**". The siPlk1-incorporated formulation (100 µL, 0.91 µM) was intravenously injected via tail vein, and the fluorescence images were taken at the time points of 30, 60, and 90 min. All the whole-body imaging experiments were carried out under identical conditions.

The in vivo therapeutic efficacy of siRNA/Ap-CS formulation was investigated using A549 (human NSCLC) xenograft tumor model. Tumor-bearing mice were randomly divided into four treatment groups. When the tumor size reached about 6 mm in diameter, different formulations, including (i) scrambled siRNA/Ap-CS, (ii) siRNA/CS, (iii) Liposome/siRNA and (iv) siPlk1/Ap-CS, were separately administrated intravenously at a dose of 100 µL (the siRNA concentration is 0.91 µM) per animal every other day. Tumor size was measured before the next injection by caliper. The tumor growth was estimated from the relative tumor volume of V/V_0, where V and V_0 indicate the tumor volume after and before treatment, respectively. The tumor volume was calculated by the formula = tumor length × (tumor width)^2/2.

For the qPCR analysis to confirm *Plk1* miRNA level, the total RNAs in 100 mg of tumor were extracted by Trizol Reagent Kit (Invitrogen). Reverse transcription of mRNA and qPCR experiments were carried out using PrimeScript™ RT reagent Kit with gDNA Eraser (Takara, Dalian, China) and TB Green™ Premix Ex Taq™ (Takara, Dalian, China), respectively. The expression level of mRNA was quantified by the $2^{-\Delta\Delta Ct}$ (threshold cycle) method.

Discussion:

As shown in Supplementary Figure 26a and 26b, when being systemically administered into tumor-bearing mice, compared with Lipo3000/siRNA and siRNA/CS, siRNA/Ap-CS does accumulate in tumor tissues with much higher efficiency. Moreover, siRNA/Ap-CS shows the higher ability to suppress tumor growth (Supplementary Figure 26c and 26d) than Lipo3000/siRNA and siRNA/CS, which is consistent with the analysis of mRNA expression level (Supplementary Figure 26e). These data demonstrate that siRNA/Ap-CS possesses the higher in vivo gene silencing efficiency.

Supplementary References

1. R. J. Kershner *et al.*, Placement and orientation of individual DNA shapes on lithographically patterned surfaces. *Nat. Nanotech.* 4, 557-561 (2009).
2. H. Ohno *et al.*, Synthetic RNA–protein complex shaped like an equilateral triangle. *Nat. Nanotech.* 6, 116-120 (2011).
3. B. Ding, R. Sha, N. C. Seeman, Pseudohexagonal 2D DNA Crystals from Double Crossover Cohesion. *J. Am. Chem. Soc.* 126, 10230-10231 (2004).
4. Y. He, Y. Chen, H. Liu, A. E. Ribbe, C. Mao, Self-Assembly of Hexagonal DNA Two-Dimensional (2D) Arrays. *J. Am. Chem. Soc.* 127, 12202-12203 (2005).
5. F. Zhang *et al.*, Self-Assembly of Complex DNA Tessellations by Using Low-Symmetry Multi-arm DNA Tiles. *Angew. Chem. Int. Ed.* 55, 8860-8863 (2016).
6. R. P. Goodman *et al.*, Rapid Chiral Assembly of Rigid DNA Building Blocks for Molecular Nanofabrication. *Science* 310, 1661-1665 (2005).
7. R. Iinuma *et al.*, Polyhedra self-assembled from DNA tripods and characterized with 3D DNA-PAINT. *Science* 344, 65-69 (2014).
8. M. Chang, C. S. Yang, D. M. Huang, Aptamer-Conjugated DNA Icosahedral Nanoparticles As a Carrier of Doxorubicin for Cancer Therapy. *ACS Nano* 5, 6156–6163 (2011).
9. H. Lee *et al.*, Molecularly self-assembled nucleic acid nanoparticles for targeted *in vivo* siRNA delivery. *Nat. Nanotech.* 7, 389-393 (2012).
10. D. Bhatia, S. Surana, S. Chakraborty, S. P. Koushika, Y. Krishnan, A synthetic icosahedral DNA-based host-cargo complex for functional *in vivo* imaging. *Nat. Commun.* 2, 339 (2011).
11. A. M. Hung *et al.*, Large-area spatially ordered arrays of gold nanoparticles directed by lithographically confined DNA origami. *Nat. Nanotech.* 5, 121-126 (2010).
12. C. Xue *et al.*, Y-Shaped Backbone-Rigidified Triangular DNA Scaffold-Directed Stepwise Movement of a DNAzyme Walker for Sensitive MicroRNA Imaging within Living Cells. *Anal. Chem.* 91, 15678−15685 (2019).

13. C. A. Hong *et al.*, Dendrimeric siRNA for Efficient Gene Silencing. *Angew. Chem. Int. Ed.* 54, 6740-6744 (2015).

14. S. J. Hurst, A. K. R. Lytton-Jean, C. A. Mirkin, Maximizing DNA Loading on a Range of Gold Nanoparticle Sizes. *Anal. Chem.* 78, 8313-8318 (2006).

15. D. Zheng *et al.*, Topical delivery of siRNA-based spherical nucleic acid nanoparticle conjugates for gene regulation. *Proc. Natl. Acad. Sci. U.S.A.* 109 (2012).

16. N. Ponnuswamy *et al.*, Oligolysine-based coating protects DNA nanostructures from low-salt denaturation and nuclease degradation. *Nat. Commun.* 8, 15654 (2017).

17. Y. Lei et al., Gold nanoclusters-assisted delivery of NGF siRNA for effective treatment of pancreatic cancer. Nat. Commun. 8, 15130 (2017).

18. L. Li *et al.*, Nucleolin-targeting liposomes guided by aptamer AS1411 for the delivery of siRNA for the treatment of malignant melanomas. *Biomaterials* 35, 3840-3850 (2014).

19. S. Yang *et al.*, Nucleolin-Targeting AS1411-Aptamer-Modified Graft Polymeric Micelle with Dual pH/Redox Sensitivity Designed To Enhance Tumor Therapy through the Codelivery of Doxorubicin/TLR4 siRNA and Suppression of Invasion. *Mol. Pharmaceutics* 15, 314-325 (2018).

20. P. P. Medina, M. Nolde, F. J. Slack, OncomiR addiction in an *in vivo* model of microRNA-21-induced pre-B-cell lymphoma. *Nature* 467, 86-90 (2010).

21. L. Xu, Y. Gao, H. Kuang, L. M. Liz-Marzan, C. Xu, miRNA-Directed Intracellular Self-Assembly of Chiral Nanorod Dimers. *Angew. Chem. Int. Ed.* 57, 10544-10548 (2018).

22. C. Wu *et al.*, A Nonenzymatic Hairpin DNA Cascade Reaction Provides High Signal Gain of mRNA Imaging inside Live Cells. *J. Am. Chem. Soc.* 137, 4900-4903 (2015).

23. H. Xing *et al.*, Selective Delivery of an Anticancer Drug with Aptamer-Functionalized Liposomes to Breast Cancer Cells *in Vitro* and *in Vivo*. *J. Mater. Chem. B* 1, 5288-5297 (2013).

24. S. N. Barnaby, A. Lee, C. A. Mirkin, Probing the inherent stability of siRNA immobilized on nanoparticle constructs. *Proc. Natl. Acad. Sci. U.S.A.* **111**, 9739-9744 (2014).

25. L. Yuan, X. Han, W. Li, D. Ren, X. Yang, Isoorientin Prevents Hyperlipidemia and Liver Injury by Regulating Lipid Metabolism, Antioxidant Capability, and Inflammatory Cytokine Release in High-Fructose-Fed Mice. *J. Agric. Food Chem.* 64, 2682−2689 (2016).

26. S. Ramaswamya *et al*., Systemic delivery of factor IX messenger RNA for protein replacement therapy. *Proc. Natl. Acad. Sci. U.S.A.* **114**, E1941-E1950 (2017).

27. F. Cardarelli *et al*., Cholesterol-Dependent Macropinocytosis and Endosomal Escape Control the Transfection Efficiency of Lipoplexes in CHO Living Cells. *Mol. Pharmaceutics* 9, 334−340 (2012).

28. P. Wu *et al*., A DNAzyme-Gold Nanoparticle Probe for Uranyl Ion in Living Cells. *J. Am. Chem. Soc.* 135, 5254−5257 (2013).

29. M. Ren *et al.*, A fast responsive two-photon fluorescent probe for imaging H_2O_2 in lysosomes with a large turn-on fluorescence signal. *Biosens Bioelectron*. 79, 237−243 (2016).

30. G. Cheng, L. Zhu, R, Mahato, Caspase-3 Gene Silencing for Inhibiting Apoptosis in Insulinoma Cells and Human Islets. *Mol. Pharmaceutics* 5, 1093–1102 (2008).

30. L. Larsen *et al*., Expression and Localization of microRNAs in Perinatal Rat Pancreas: Role of miR-21 in Regulation of Cholesterol Metabolism. *Plos One*, 6, e25997 (2011).
