# Supplementary material for: Programmably tiling rigidified DNA brick on gold nanoparticle as multi-functional shell for cancer-targeted delivery of siRNAs
Source: Nat Commun. 2021 May 18;12:2928. doi: 10.1038/s41467-021-23250-5 (PMC8131747; doi:10.1038/s41467-021-23250-5)
Supplement: Supplementary file 3 — Reporting Summary [file 41467_2021_23250_MOESM3_ESM.pdf]

nature research

Corresponding author(s): Zai-Sheng Wu

Last updated by author(s): Mar 14, 2021

Reporting Summary

Nature Research wishes to improve the reproducibility of the work that we publish. This form provides structure for consistency and transparency in reporting. For further information on Nature Research policies, see our Editorial Policies and the Editorial Policy Checklist.

Statistics

For all statistical analyses, confirm that the following items are present in the figure legend, table legend, main text, or Methods section.

n/a	Confirmed	
☐	☒	The exact sample size (*n*) for each experimental group/condition, given as a discrete number and unit of measurement
☐	☒	A statement on whether measurements were taken from distinct samples or whether the same sample was measured repeatedly
☐	☒	The statistical test(s) used AND whether they are one- or two-sided<br>*Only common tests should be described solely by name; describe more complex techniques in the Methods section.*
☐	☒	A description of all covariates tested
☐	☒	A description of any assumptions or corrections, such as tests of normality and adjustment for multiple comparisons
☐	☒	A full description of the statistical parameters including central tendency (e.g. means) or other basic estimates (e.g. regression coefficient) AND variation (e.g. standard deviation) or associated estimates of uncertainty (e.g. confidence intervals)
☐	☒	For null hypothesis testing, the test statistic (e.g. *F*, *t*, *r*) with confidence intervals, effect sizes, degrees of freedom and *P* value noted<br>*Give P values as exact values whenever suitable.*
☒	☐	For Bayesian analysis, information on the choice of priors and Markov chain Monte Carlo settings
☐	☒	For hierarchical and complex designs, identification of the appropriate level for tests and full reporting of outcomes
☐	☒	Estimates of effect sizes (e.g. Cohen's *d*, Pearson's *r*), indicating how they were calculated

Our web collection on statistics for biologists contains articles on many of the points above.

Software and code

Policy information about availability of computer code

Data collection	Bio Rad CFX Manager 3.0, Image Lab 5.0, Leica Microsystems CMS GmbH 1.1.0, Nano Scope Analysis 1.70, Flow Jo 7.6.1, and IVIS Living image 4.4 were used to collect data.
Data analysis	GraphPad Prism 8, DAS version 3.2.2, and image j 2.0 were used to analyze date.

For manuscripts utilizing custom algorithms or software that are central to the research but not yet described in published literature, software must be made available to editors and reviewers. We strongly encourage code deposition in a community repository (e.g. GitHub). See the Nature Research guidelines for submitting code & software for further information.

Data

Policy information about availability of data

All manuscripts must include a data availability statement. This statement should provide the following information, where applicable:

- Accession codes, unique identifiers, or web links for publicly available datasets
- A list of figures that have associated raw data
- A description of any restrictions on data availability

The date are available from the corresponding authors upon reasonable request. Source date are provided as a Source Date file. Source date are provided with this paper.

Field-specific reporting

Please select the one below that is the best fit for your research. If you are not sure, read the appropriate sections before making your selection.

☒ Life sciences ☐ Behavioural & social sciences ☐ Ecological, evolutionary & environmental sciences

For a reference copy of the document with all sections, see nature.com/documents/nr-reporting-summary-flat.pdf

Life sciences study design

All studies must disclose on these points even when the disclosure is negative.

Sample size	The sample size was determined at least three times by related reports and similar experiments (eg. fluorescence scanning in Fig.3, Supplementary Figure 5, Supplementary Figure 9 et al; PCR in Fig.6, Supplementary Figure 19, Supplementary Figure 24; Confocal fluorescence imaging in Fig 5. Supplementary Figure 13, Supplementary Figure 14, Supplementary Figure 18 et al; animal experiment in Fig. 4, Fig. 7, Supplementary Figure 8, Supplementary Figure 26 et al).
Data exclusions	There is no data exclusions.
Replication	All attempts in replication were successful and the experiment such as fluorescence scanning, PCR, Confocal fluorescence imaging, animal experiment et al were repeated at least three times independently.
Randomization	All the groups in this study are random.
Blinding	The investigators were blinded to group allocation during data collection and analysis.

Reporting for specific materials, systems and methods

We require information from authors about some types of materials, experimental systems and methods used in many studies. Here, indicate whether each material, system or method listed is relevant to your study. If you are not sure if a list item applies to your research, read the appropriate section before selecting a response.

Materials & experimental systems

n/a	Involved in the study	
☐	☒	Antibodies
☐	☒	Eukaryotic cell lines
☒	☐	Palaeontology and archaeology
☐	☒	Animals and other organisms
☒	☐	Human research participants
☒	☐	Clinical data
☒	☐	Dual use research of concern

Methods

n/a	Involved in the study	
☒	☐	ChIP-seq
☐	☒	Flow cytometry
☒	☐	MRI-based neuroimaging

Antibodies

Antibodies used	Anti-Plk1 (208G4) Rabbit mAb #4513, GAPDH (D16H11) XP® Rabbit mAb #5174, Anti-rabbit IgG (HRP-linked Antibody) #7074
Validation	Anti-Plk1 antibody: Description: Rabbit monoclonal, Application: WB, IP, IHC, Reactivity: Rat, Human, Monkey<br>GAPDH antibody: Description: Rabbit monoclonal, Application: WB, IHC, IF, Reactivity: Mouse, Rat, Human, Monkey<br>Anti-IgG antibody: Description: Rabbit monoclonal, Application: WB

Eukaryotic cell lines

Policy information about cell lines

Cell line source(s)	MCF-7, HeLa, A549, LO2 were obtained from Institute of Biochemistry and Cell Biology, Chinese Academy of Science (Shanghai, China). INS-1E cells was obtained from Shanghai hongshun Biotechnology Co., Ltd.
Authentication	All the cells in this study were obtained from standard commercial sources authentication or isolated according to reported researches. The STR profiling of cells have been performed.
Mycoplasma contamination	All the cell lines were tested negative for mycoplasma contamination.
Commonly misidentified lines (See ICLAC register)	None

Animals and other organisms

Policy information about studies involving animals; ARRIVE guidelines recommended for reporting animal research

Laboratory animals	Nude mouse: BALB/c, female 3-5week, KM mouse: female 4-6week, cultured at an ambient temperature of 25 °C and humidity of 40–60% under a 12-h light/dark cycle.
Wild animals	None
Field-collected samples	The animals were kept in a cage and cultured at 25 degrees centigrade, and no field collected samples were used.
Ethics oversight	All animal protocol procedures approved by the Institutional Animal Care and Use Committee (IACUC) of Fuzhou University (approval number: SYXK-2019-0007).

Note that full information on the approval of the study protocol must also be provided in the manuscript.

Flow Cytometry

Plots

Confirm that:

- [x] The axis labels state the marker and fluorochrome used (e.g. CD4-FITC).
- [x] The axis scales are clearly visible. Include numbers along axes only for bottom left plot of group (a 'group' is an analysis of identical markers).
- [x] All plots are contour plots with outliers or pseudocolor plots.
- [x] A numerical value for number of cells or percentage (with statistics) is provided.

Methodology

Sample preparation	After incubating in siPlk-incorporated formulation solution at 37 °C for 4 h, the cells were treated with trypsinization, followed by flow cytometric analysis on Becton Dickinson (BD) multiparametric fluorescence-activated cell sorting (FACS) Aria III cell sorter.
Instrument	BD FACSAria III
Software	Flow Jo 7.6.1
Cell population abundance	Cells without incubating with siPlk-incorporated formulation solution were used as control, and the preliminary FSC/SSC gates were established according to the characteristic population of control. The abundance of cells incubated with siPlk-incorporated formulation were obtained by the gate of control group.
Gating strategy	Cells without incubating with siPlk-incorporated formulation solution were used as control, and the preliminary FSC/SSC gates were established according to the characteristic population of control. The control group as negative population, the fluorescence exceeded the control group as positive population.

- [x] Tick this box to confirm that a figure exemplifying the gating strategy is provided in the Supplementary Information.
